# Supplementary material for: Experiences using the poststroke checklist in Sweden with a focus on feasibility and relevance: a mixed-method design
Source: BMJ Open. 2019 May 9;9(5):e028218. doi: 10.1136/bmjopen-2018-028218 (PMC6528008; doi:10.1136/bmjopen-2018-028218)
Supplement: Author's manuscript [file bmjopen-2018-028218.draft_revisions.pdf]

BMJ Open is committed to open peer review. As part of this commitment we make the peer review history of every article we publish publicly available.

When an article is published we post the peer reviewers' comments and the authors' responses online. We also post the versions of the paper that were used during peer review. These are the versions that the peer review comments apply to.

The versions of the paper that follow are the versions that were submitted during the peer review process. They are not the versions of record or the final published versions. They should not be cited or distributed as the published version of this manuscript.

BMJ Open is an open access journal and the full, final, typeset and author-corrected version of record of the manuscript is available on our site with no access controls, subscription charges or pay-per-view fees (http://bmjopen.bmj.com).

If you have any questions on BMJ Open's open peer review process please email info.bmjopen@bmj.com

BMJ Open

Experiences using the Post-Stroke Checklist in Sweden with a focus on feasibility and relevance: a mixed-method design

Journal:	*BMJ Open*
Manuscript ID	bmjopen-2018-028218
Article Type:	Research
Date Submitted by the Author:	27-Nov-2018

Complete List of Authors:	Kjörk, Emma K; University of Gothenburg, Institute of Neuroscience and Physiology, Department of Clinical Neuroscience<br>Carlsson, Gunnel; University of Gothenburg, Institute of Neuroscience and Physiology, Department of Clinical Neuroscience<br>Sunnerhagen, Katharina; University of Gothenburg, Institute of Neuroscience and Physiology, Department of Clinical Neuroscience<br>Lundgren-Nilsson, Asa; Gothenburg University, Institute of Neuroscience and Physiology, Department och Clinical Neuroscience

Keywords:	long-term care, Feasibility, Focus groups, Follow-up, Standard of Care, Stroke < NEUROLOGY

SCHOLARONE™ Manuscripts

Experiences using the Post-Stroke Checklist in Sweden with a focus on feasibility and relevance: a mixed-method design

Emma K Kjörk* OT, MS[1, 2], Gunnel Carlsson OT, PhD[1], Katharina S Sunnerhagen MD, PhD, Prof[1] and Åsa Lundgren-Nilsson OT, PhD[1]

[1]Institute of Neuroscience and Physiology, Dep of Clinical Neuroscience, Sahlgrenska Academy at University of Gothenburg, Gothenburg, Sweden
[2]Department of Occupational Therapy and Physiotherapy, Sahlgrenska University Hospital, Gothenburg, Sweden

* Corresponding author:
Emma K Kjörk
Email address: emma.kjork@neuro.gu.se
Telephone: +46313422803
Postal address:
Institute of Neuroscience and Physiology
Dep of Clinical Neuroscience, Rehabilitation medicine
Per Dubbsgatan 14, fl. 3, 413 45 Gothenburg, Sweden

Word count Abstract: 286 words
Word count Main manuscript: 3769 words
Number of tables: 3
Number of figures: 3

For peer review only - http://bmjopen.bmj.com/site/about/guidelines.xhtml

Experiences using the Post-Stroke Checklist in Sweden with a focus on feasibility and relevance: a mixed-method design

ABSTRACT

Objective The wide range of outcomes after stroke emphasises the need for comprehensive long-term follow-up. The aim of this study was to evaluate how persons with stroke and health professionals perceive the use of the Post-Stroke Checklist (PSC), with a focus on feasibility and relevance.

Design An exploratory design with a mix of qualitative and quantitative methods.

Setting Outpatient care at a university hospital and primary care centres in western Sweden.

Participants Forty-six consecutive patients (median age, 70; range, 41–85; 13 women) and ten health professionals (median age 46; range, 35-63; 7 women).

Results Most patients (87%) had one or more problems identified by the PSC. The most common problem areas were life after stroke (61%), cognition (56%), mood (41%), and activities of daily living (39%). Three organisational themes emerged from the focus group discussions. The perception of *the content and relevance of* the PSC was that common post-stroke problems were covered but that unmet needs still could be missed. Identifying needs was facilitated when using the *PSC as a tool for dialogue.* The dialogue between the patient and caregiver as well as stroke competence was perceived as important. The PSC was seen as *a systematic routine and a base for egalitarian follow-up*, but participants stressed consideration given to each individual. Addressing identified needs and meeting patient expectations were described as challenging given available healthcare services.

Conclusions The PSC is a feasible and relevant tool to support egalitarian follow-up and identify patients who could benefit from targeted post-stroke interventions. Stroke competence, room for dialogue, and caring for identified needs emerged as important issues to consider when using the PSC. The PSC can facilitate patients in expressing their needs, enhancing their ability to participate in decision-making.

For peer review only - http://bmjopen.bmj.com/site/about/guidelines.xhtml

Keywords Stroke, Rehabilitation, Standard of care, Feasibility, Focus groups, Follow-up

Strengths and limitations (bullet points)

- A strength of this study is having patients as partners throughout the project, from the translation process to participation in the focus groups.
- The mix of methods made it possible to explore the feasibility of the PSC from different perspectives and at different levels compared to previous research.
- A limitation is the lack of data collected from persons with severe stroke, although this lack is typical of this naturalistic design and representative for the Swedish stroke population.
- A study strength is the heterogeneous population from different outpatient settings, including a range of ages, stroke characteristics, and education levels, and health professionals with a range of experience in stroke care.
- The study was conducted in a Swedish context, and the transferability of the findings in other cultural contexts is not known.

For peer review only

INTRODUCTION

Post-stroke impairments often have long-term negative consequences for social relationships, dependence in daily life, and quality of life.[1] Perceived unfulfilled rehabilitation needs and changes in functioning during the first year after stroke[2 3] indicate the need for systematic long-term follow-up. Furthermore, a subtle decline in cognition as well as emotional problems[4 5] can easily be overlooked, leading to difficulty in accessing healthcare services.[6 7]

The adaptation process after stroke is long. In later phases, the focus changes from the stroke itself to more familiar aspects of daily life,[8] which may not be sufficiently targeted in current practice. Concordance is poor between perceived problems and problems detected by standardised assessments. Accordingly, dialogue should complement assessments to ensure that health services are based on patient needs,[9] in line with a person-centred approach.[10]

The Post-Stroke Checklist (PSC)[11] was developed to identify long-term care needs and facilitate referrals.[11 12] Although the PSC has been found to be feasible and useful,[12-14] knowledge is lacking about the perspectives of patients and health professionals (HPs) regarding its use in the Swedish health care context. The aim of this study was to evaluate how persons with stroke and HPs perceive the use of the PSC, with a focus on feasibility and relevance.

METHODS

Study design

The study has an exploratory design. To capture the feasibility of using the PSC, we combined it with a satisfaction questionnaire and focus group discussions,[15 16] in line with guidelines for complex interventions.[17] This study is part of a validation and cultural adaptation process[18] of the PSC in Sweden (figure 1). By combining data collection methods, we expected to gain a deeper understanding of using the PSC as a tool to structure follow-up. The underpinning methodology in the focus groups is based on social constructivism.[16] Consolidated criteria for reporting qualitative research (i.e., COREQ guidelines)[19] were followed for reporting qualitative data.

[Insert figure 1 near here]

For peer review only - http://bmjopen.bmj.com/site/about/guidelines.xhtml

Patient and public involvement

This study explores how persons with stroke experience the PSC. People from the Swedish Stroke Association (patient association) were involved in the translation process, the pilot testing of the interview guide, and focus group discussions and have been given a presentation of preliminary results.

Participants

Participants were consecutively recruited while at a clinical visit in primary care or stroke specialised outpatient care at a university hospital, February 2015 to October 2015. The inclusion criterion was having had a stroke, regardless of the time of onset. The number of patients included was in accordance with a previous study[12] and principles for cross-cultural adaptation suggesting approximately 40.[18] Patients were excluded if cognitive impairment or insufficient knowledge of Swedish would have made the response to the PSC items unreliable. HPs from different clinics were invited to participate and selected to represent different professions. A purposive sampling was used to achieve heterogeneity and homogeneity in the focus groups.[15 16] Written informed consent was obtained from all participants, and the regional ethical review board in Gothenburg approved the study (no. 521-14).

Data collection

The PSC[11] has 11 items and is intended to identify post-stroke problems. It was developed by a multi-professional group of stroke experts, according to a Delphi process. Problem areas were chosen for having the greatest impact on patient quality of life that could be addressed with evidence-based interventions. The PSC includes secondary prevention, activities of daily living (ADLs), mobility, spasticity, pain, incontinence, communication, mood, cognition, life after stroke, and relationship with family. A response scale includes 'yes' and 'no' and recommended referrals adjacent to each problem area.[11]

Data collection was conducted in outpatient clinical facilities in two steps. For step 1, the HP administered the PSC to patients at a regular clinical visit (proxy responses were allowed). No additional training was given beyond the general instructions on the PSC. Participants were asked to reflect on the usefulness of the PSC as the basis for focus group discussions. Patients and HPs assessed satisfaction with the PSC after each visit through a satisfaction questionnaire with questions analogous to those used in a previous study.[12] The answers were rated on a Likert scale of 1–5, where 1 indicated not satisfied and 5 completely

For peer review only - http://bmjopen.bmj.com/site/about/guidelines.xhtml

satisfied. To ensure anonymity, patient responses were collected in an envelope. Demographic data (time since index stroke, age, sex), time to administration of the PSC, and HP profession were registered. Of the patients who gave informed consent, additional patient characteristics (such as type of stroke, aphasia, ADL dependency) were collected retrospectively from their medical records.

For step 2, the staff invited all participants during the follow-up to join in a focus group discussion. A set time of approximately 1-2 months was given between the visit and the focus group discussions. The first author (EK) telephoned participants willing to participate and sent them the study information letter, time for appointment, and a copy of the PSC. In total, four focus groups were conducted using a question guide.[15] Each focus group met once for approximately 1.5 hours. The meeting was recorded and transcribed verbatim. Initially in the focus groups, the importance of bringing up different opinions was emphasised. At the end, an oral overview was presented to ensure that participant contributions were as they intended.

Data analysis

Data gathered from PSC items, questionnaires, and demographic information were analysed using descriptive statistics with Statistical Package for the Social Sciences version 24. Data gathered from focus groups were analysed following Kreuger.[15] The aim of the analysis process was to describe the participants' perceptions and experiences based on the aim of the study.

Immediately after each focus group discussion, a written summary was created. The transcripts were coded using the computer software NVivo. First, the transcripts were read in their entirety to allow familiarity with the content as a whole, and discussions relevant to the aim were identified. Second, transcripts were systematised into categories based on similarities and differences in the discussions. Third, a descriptive summary was made for each category, adhering as closely as possible to the content of the raw data. Finally, these summaries in combination with selected quotes served as the basis for the interpretation and presented a deeper insight into the findings. In the analysis process, identified patterns were compared and contrasted across all four groups, resulting in an overarching thematic structure (for examples of the coding tree and themes see table 1 and figure 3). Quotations that showed the ongoing discussions[16] were selected to illuminate the results. Based on sampling strategies[20] and when similar discussions recurred in all groups[15], data gathering stopped.

The first author (PhD student, OT, woman) was the moderator and performed most of the analysis. Multiple coding, continuous interpretation of data, and discussion of the

For peer review only - http://bmjopen.bmj.com/site/about/guidelines.xhtml

emerging themes were completed together with the second author (PhD, OT, woman) to ensure accuracy of the analysis. The first author had knowledge about the study topic and the second author in qualitative methods. Both have conducted interviews previously. The third author (PhD, MD, woman) and last author (PhD, OT, woman) contributed with knowledge concerning revising and refining the themes. All authors have at least 20 years of experience in stroke rehabilitation.

Table 1. Examples illustrating the coding tree.

Quote	Code	Subtheme
P4:"It has a lot to do with the competence of the person who's asking the questions so they can do the thinking to squeeze it all in" (Group 1 Patients)	The professionals competence and reasoning	The importance of HPs' competence in stroke and communication skills for capturing patient needs
P2: So, I think it has just been positive, and it is also done so quickly.<br>P1: That's also a positive.<br>P2: Yeah, it's fast, but you can also develop it as much as you want. But asking the questions doesn't take too long.<br>Moderator: Is it quick?<br>P1: Yes. It also depends on what answers you get.<br>P2: Yes.<br>(Group 4. Health professionals)	The administration of the PSC can be adapted, quickly done or more in-depth	The PSC supports continuity and referrals but depends on available resources

For peer review only

RESULTS

Study group

The PSC was used in connection with a clinical visit in 46 patients. All patients lived in their homes. Most of them (65%) had experienced a stroke within 3 months at enrolment. The median time for hospitalisation was 8 days. Stroke severity at stroke onset was mild, with a median of 2 according to National Institutes of Health Stroke Scale. Table 2 shows the characteristics of the participants and the focus groups.

For peer review only - http://bmjopen.bmj.com/site/about/guidelines.xhtml

Table 2. Characteristics of patients and health professionals in the clinical outpatient visits and the focus group discussions. Data are presented as number of persons (n) or median and range.

**Patients**	Clinical visit (n=46)	Focus group 1 (n=4)	Focus group 2 (n=6)
Primary care, rural		x	
Specialized care, urban			x
Age at inclusion	70 (41-85)	71 (58-78)	74 (45-76)
Sex, male	33	4	5
Education			
Mandatory	20	1	4
High School	13	1	1
University	8	2	1
Months since stroke	3 (1-84)	20 (3-84)	3 (1-6)
Working at stroke onset (yes)	13	2	1
Length of hospitalization, (days)	8 (2-120)	11 (5-82)	8 (4-11)
History of stroke (yes)	9	1	3
Stroke characteristics			
Ischemic/Hemorrhagic	36/5	4/0	4/2
Right/ left/ posterior/ bilateral	19/16/5/2	3/1/0/0	3/2/1/0
NIHSS	2 (0-16)	4 (3-10)	2 (1-6)
Aphasia (yes)	9	0	1
Neglect (yes)	4	1	0
At discharge			
ADL independency (yes)	34	3	6
Wheel-chair use (yes)	4	1	0
**Health Professionals**	Clinical visit (n=10)	Focus group 3 (n=4)	Focus group 4 (n=4)
Age	46 (35-63)	43 (37-46)	46 (35-55)
Primary care, rural			x
Specialized care, urban		x	
Sex, male	3	0	1
Nurse/ OT/ Physician	4/1/5	3/0/1	0/1/3
Stroke experience (years)			
≤5/ 5-10/ 10	2/2/6	0/1/3	2/1/1

Abbreviations: NIHSS=National Institutes of Health Stroke Scale, ADL= Activities of Daily Living, OT= Occupational Therapist.

(Missing data from medical records (n=4).

For peer review only

For peer review only - http://bmjopen.bmj.com/site/about/guidelines.xhtml

Feasibility of the PSC

Forty patients (87%) had one or more problems identified by the PSC (figure 2). ‘Life after stroke’ was most common (61%), followed by cognition (56%) and mood changes (41%). A median of four problem areas per patient (range, 0–9; interquartile range [IQR], 1–5) was identified; the median in specialised care was 3 (IQR, 1–5), and in primary care, it was 4 (IQR, 1–5). Only six (13%) patients reported no problems. Most patients (70%) acknowledged having received information about secondary prevention. Referrals were registered in eight cases, slightly more often in primary care (n=6) than in specialised care (n=2). The time taken to administer the PSC was ≤15 minutes for 52%, ≤30 minutes for 43%, and ≥45 minutes for 5%.

[Insert figure 2 near here]

Four focus groups were conducted, and their characteristics are shown in table 2. One woman dropped out because of a scheduled medical examination. The focus group discussions revealed that the PSC structure in combination with room for dialogue could support egalitarian follow-up and identification of needs. A main theme and three organisational themes emerged in these discussions (figure 3).

[Insert figure 3 near here]

The content and relevance of the PSC

Item relevance

The items included in the PSC were considered relevant to all groups. Because stroke affects persons differently, participants found it valuable that the PSC covers a broad spectrum of problems, although not all problems are relevant to every person with stroke.

The PSC ensures coverage of important areas, but excluded areas could be missed

Both patients and HPs stated that some issues might be overlooked if not specifically stated in the PSC; such as nutrition, sexuality, and driving. The HPs discussed the appropriate amount of problem areas in the PSC. They wanted more areas yet preferred the checklist to be short and complemented by profession-specific assessments when needed. Some participants appreciated the recurring phrase “since your stroke”, but others preferred to hear/say it once at the beginning of the visit. Patients perceived the PSC as easy to understand, while HPs

For peer review only - http://bmjopen.bmj.com/site/about/guidelines.xhtml

expressed a concern about misunderstandings, especially for the items 'secondary prevention' and 'spasticity'.

The PSC as a tool for dialogue

Patients and HPs both emphasised the need for dialogue to create mutual understanding. HPs described that knowledge and experience affected their ability to detect problems, while patients described differences in their ability to communicate problems.

Dialogue facilitates patients in expressing needs and engenders feelings of being cared for

Patients said that the PSC questions facilitated dialogue, leading to a greater likelihood that important areas would be elucidated and discussed. Memory problems, lack of initiative, fatigue, or being less talkative were mentioned as barriers to dialogue that the PSC could address. The PSC gave clear direction for the structure of the dialogue and accordingly facilitating identification of problems. Nevertheless, using the PSC in combination with dialogue was seen as important. Patients stated that they might need time for consideration before answering the PSC questions, time that was often not given within the limits of the visits.

Generally, participants thought patients should have the opportunity to talk to a professional about stroke-related concerns and stated that the PSC could facilitate this exchange. Patients expressed that the PSC covers areas centring on them as a person, which made them feel cared for. The understanding of the HPs was that relatives often complemented information concerning problems that patients might neglect or forget to mention:

> *P4: "I think there is a great deal of importance to how much you are affected by the stroke. The more you are affected, the harder it is to think about the different facets of it"* (the areas in the PSC). (Strongly agreed upon)
> (Group 1. Patients)

The importance of HPs' competence in stroke and communication skills for capturing patient needs

To ensure that problems would be fully addressed, a professional's competence in stroke was seen as key by the participants. HPs feared that lack of stroke competence might lead to

For peer review only - http://bmjopen.bmj.com/site/about/guidelines.xhtml

problems going unrecognised. The PSC was seen as an asset as well as a barrier to dialogue. If too much focus was placed on posing the questions, participants experienced a decreased interaction. Sensitivity from the HPs and use of additional questions was seen as essential:

> *P7: "No, there's only benefits, but it depends on how you use it, and if the staff think it is meaningful, so it's not just checked off. Rather that you have the opportunity to cover the things that each point is actually about".*
> *P8: "Yeah, all the stuff that's crazy [difficulties after the stroke], follow up that stuff."*
> *Moderator: "That you have time to follow up what is included in the point? Can you elaborate on that?"*
> *P7: "Yes, it's the topic this question is about, that you can elaborate on if you want to (...) the person with the checklist shouldn't be bound to it 100% and slavishly follow it, but understand signals from the patient and talk more broadly and connect it to the other things that depend upon it". (agreement)*
> *P9: "Absolutely".*
> *P7: "Otherwise, it just becomes mechanical; you can't be just like a computer asking questions".*
> (Group 2. Patients)

There were conflicting opinions about how to apply the questions in the PSC. Experienced HPs preferred to use it as a supplement for memory within a free dialogue. In contrast, inexperienced HPs perceived the specific questions as good to assume and a basis for leading into other related concerns (i.e., fatigue):

> *P5: "It's more about if you think that you should use standardised things for everyone, even for primary healthcare/outpatient care, I don't think that really works".*
> *P6: "Exactly, that's the question, should you use the checklist just as it is, or should you use it for your own part and remember. That's the thing, because you can then approach each patient differently and get it all. But asking the exact same questions for each patient, I agree, that's really hard to do".*
> (Group 3. HPs)

For peer review only - http://bmjopen.bmj.com/site/about/guidelines.xhtml

For peer review only

PSC as a systematic routine and a basis for egalitarian follow-up

The PSC as a shared knowledge base to be individualised for each patient

The PSC was considered to increase knowledge about stroke and secure an egalitarian follow-up, especially for inexperienced HPs and patients with limited ability to express their needs. Even when the PSC was used, lack of stroke competence and limited knowledge about opportunities for referrals were perceived as an obstacle to egalitarian follow-up. One suggestion was to add local referral opportunities and access to scientific references in conjunction with the PSC. Factors such as comorbidities and time since stroke must be taken into account because they could affect responses to PSC items.

The PSC supports continuity and referrals but depends on available resources

Participants addressed the need for regular follow-ups and considered the PSC to be a useful tool and basis for referrals. The use of the PSC was seen as a rapid way to cover the problem areas if only the questions were used, but when supplementary questions were needed, the time need also increased. One concern, especially among the physicians, was the time taken to administer the PSC in addition to their ordinary routines. HPs emphasised that the use of the PSC should be beneficial for the patients and in accordance with time limits and referral opportunities. Some considered it a bit rigid to go through all items if the patient experienced no problems, although it was observed that it could be completed quickly.

To enable preparation beforehand and make visits more time-efficient, a patient version of the PSC was proposed. Participants strongly emphasised that problems identified by the PSC should lead to appropriate intervention and not only an evaluation of current status:

> *P11: "The risk is you might get a false sense of security though. So, someone has asked the question, and I have answered "yes" to this question; so I then expect something to happen".*
>
> (agreement from the others)
>
> *P11: "It's like, that's what decides the quality of what happens with the measures (...) It should end with me knowing how this information is taken and handled, what happens now. Not just that you do it and then that's great. (mumbles) Is it like statistics or what?"*
>
> (Group 1. Patients)

For peer review only - http://bmjopen.bmj.com/site/about/guidelines.xhtml

Lack of opportunities for interventions as well as knowledge gaps were expressed by HPs as difficulties in meeting these expectations. A specific dimension of this problem was mentioned as leading to a risk of avoidance of discussing certain items.

Table 3. Evaluation of the use of Post-Stroke Checklist (PSC) based on satisfaction ratings (likert 1-5) by patients and health professionals.

Satisfaction with:	Patients (n=46) median (IQR)	Health professionals (n=10) median (IQR)
Overall assessment where PSC was used	5 (4-5)	4 (3-4)
Identification of needs	5 (4-5)	-
Identification of need (for each patient)	-	3 (3-4)
Confidence in receiving support	5 (4-5)	-
Guidance for referrals and treatment	-	3 (2-4)

Abbreviations: IQR= interquartile range

By combining the results derived by different methods, additional aspects of the analysis can be demonstrated. The patients evaluated the satisfaction with the PSC as high (table 3). In addition, the focus group analyses gave insights into a wide range of factors that affect satisfaction, and its feasibility was exemplified by the importance of dialogue (figure 3). HP satisfaction with the PSC varied among patients (table 3). Participants perceived it as important to adapt the use of the PSC to individual aspects. Some differences regarding individual prerequisites are displayed in table 2, and others are mentioned in the qualitative analyses. HP stroke experience varied, especially in the primary care settings (table 2). In the focus group discussions, competence in stroke was perceived as important if subtle problems were to be properly recognised.

DISCUSSION

The PSC is a relevant and feasible tool to identify patients who can benefit from targeted interventions, as noted by persons with stroke and HPs. The original purpose of the PSC was to be an easy-to-use tool to detect post-stroke problems, as well as a support for guiding referrals.[11] This study brings out an awareness about how follow-up through the PSC could be enhanced by user perceptions and suggested strategies. This knowledge could add important insights when implementing the PSC in line with the World Stroke Organization recommendations. The focus group discussions raised issues concerning prerequisites when

For peer review only - http://bmjopen.bmj.com/site/about/guidelines.xhtml

using the PSC. These include HP stroke competence, room for dialogue, and how the identified needs were addressed.

The wide range of post-stroke problems identified in the present study demonstrated the relevance of the PSC, with a median of four problems per patient. Of note, reported problems on specific PSC items differ considerably; 'life after stroke', cognition, and continence vary when comparing among countries.[12-14] Based on issues raised in the focus group discussions, likely causes of these discrepancies in reported problems could be HP stroke competence, opportunity for a dialogue, and time limits on the administration of the PSC. Furthermore, cultural aspects, differences in healthcare systems, and follow-up services could affect the results. Nevertheless, the wide range of identified problems alongside participant perceptions in this study stresses the relevance of using the PSC in clinical practice. The long-term consequences after stroke emphasise the need for a comprehensive long-term follow-up with a multi-domain approach.[1 3 21-23]

The present study provides a deeper understanding of how the PSC structure could support patients in expressing their needs. Problems with communication and comprehension are common after stroke,[5] which influences decision-making during follow-up. Participation in decision-making requires health literacy, i.e., the competence to understand information and a capacity to argue for one's needs in relation to appropriate interventions.[7] In this study, despite perceptions that the PSC questions were easy to understand, dialogue was found to be crucial. Participants raised concerns about problem areas that could be missed depending on how the PSC was used. Results from using the PSC in the UK and Singapore[12] indicate that several problem areas could be indirectly identified. Awareness of the complexity of need identification underscores the role of the HP when using the PSC. Even if no unmet need is reported, a person can still identify as living with residual impairments and perceived problems in engaging in activities.[24] Participants expressed that identification of needs could be enhanced if more time were allowed for consideration of these needs and for additional questions; another help would be the opportunity to fill in the PSC beforehand. Current findings stress HP stroke competence and the need to make space for dialogue when administering the PSC to support needs identification and decision-making.

The results from the present study highlight the dialogue between the patient and HP, which is central in health care.[9 10 25] Experienced HPs argued that they could cover most topics using open-ended questions. In contrast, others emphasised the value of articulating the PSC questions literally. To enable investigation of specific areas, closed questions can be important[26] and facilitate situations involving patients with communication difficulties.

For peer review only - http://bmjopen.bmj.com/site/about/guidelines.xhtml

However, the PSC instructions do not hinder its use in a looser way as long as all areas are captured. The result shows that patients can benefit from a clear structure when the PSC is used. Participants in all focus groups agreed on the benefits of going through the areas in the PSC in a way that ensures identification of unmet needs. In addition, using the PSC in combination with dialogue supports the patients' capacity to communicate their needs. A narrative communication, along with signs of problems, gives the HP a foundation for planning care together with the patient and creates conditions for patients to make appropriate health decisions.[10] The importance of clarifying the prerequisites for follow-up, as well as creating a plan to take care of identified needs, was noted in the focus group discussions.

Strengths and limitations

A strength of this study is that patients were partners throughout the project, from the translation process to participation in the focus group discussions. In addition, the mix of methods made it possible to explore the feasibility of the PSC at different levels compared to previous research. Few persons in this population had lived with their stroke for a long time, and only one of them had a severe stroke, which might have affected which problems were identified. This naturalistic design in an ordinary outpatient context, however, is representative of the Swedish population, where the majority have mild stroke. The time (1-2 months) between the follow-up visit where the PSC was used and the focus group discussion could be a risk for recall bias. However, the focus group methodology enabled exploring a range of opinions of people across groups, and together, the participants contributed to rich discussions. A strength of the study is the heterogeneous sample from different outpatient settings, with patients having a range of ages, stroke characteristics, and education levels, and HPs with a range of professional roles and experience in stroke. To strengthen the transferability of the findings, a comprehensive description of the study context, participant characteristics, data collection, and analysis process are included in the methods section. Because the World Stroke Organization recommends using the PSC globally, these results contribute to a deeper understanding of its feasibility that can be transferable to other countries, although consideration must be given to cultural and contextual factors.

Conclusions

The PSC is a feasible and relevant tool to support egalitarian follow-up and identify patients who can benefit from targeted interventions after stroke. Stroke competence, room for dialogue, and addressing identified needs were raised as important issues to consider when

For peer review only - http://bmjopen.bmj.com/site/about/guidelines.xhtml

using the PSC. The PSC can facilitate patients in expressing their needs, enhancing their ability to participate in decision-making.

Acknowledgements

The authors thank the health professionals and patients, for sharing their experiences. The authors also thank Dr Kate Bramley-Moore for editing and translation help.

Author contribution

EK, ÅL-N, and KSS contributed to the design of the study. EK conducted the interviews and analyzed the data together with GC, involving KSS and ÅL-N in the final stages of the analysis. EK wrote the first version of the manuscript, which was reviewed by GC, ÅL-N KSS. All four authors contributed to and approved the final manuscript.

Funding

The study was supported by grants from the Swedish state under the agreement between the Swedish government and the county councils, the ALF agreement (ALFGBG-718711); the Swedish Stroke Association; the Local Research and Development Board for Gothenburg and Södra Bohuslän; the Swedish Heart and Lung foundation; the Swedish Brain Foundation; an unconditional grant from Allergan; the Hjalmar Svenssons Foundation; Neurological Foundation; Rune and Ulla Amlövs Foundation; Per-Olof Ahls Foundation; and John and Brit Wennerströms Foundation.

Competing interests

None declared.

Data sharing

Due to ethical restrictions, data are available upon request. Researchers can submit requests for data to the authors (contact: ks.sunnerhagen@neuro.gu.se). Complete data from interviews cannot be made publicly available for ethical and legal reasons, according to the Swedish regulations http://www.epn.se/en/start/regulations/. Public availability would compromise participant privacy or confidentiality. Upon request a list of condensed meaning units or codes can be made available after removal of information that may risk the confidentiality of the participants. To access data please contact the first author: (emma.kjork@neuro.gu.se).

For peer review only

REFERENCES

1. Crichton SL, Bray BD, McKevitt C, et al. Patient outcomes up to 15 years after stroke: survival, disability, quality of life, cognition and mental health. *J Neurol Neurosurg Psychiatry* 2016;87(10):1091-8. doi: 10.1136/jnnp-2016-313361

2. Ullberg T, Zia E, Petersson J, et al. Changes in functional outcome over the first year after stroke: an observational study from the Swedish stroke register. *Stroke* 2015;46(2):389-94. doi: 10.1161/STROKEAHA.114.006538

3. Ullberg T, Zia E, Petersson J, et al. Perceived Unmet Rehabilitation Needs 1 Year After Stroke: An Observational Study From the Swedish Stroke Register. *Stroke* 2016;47(2):539-41. doi: 10.1161/STROKEAHA.115.011670

4. Carlsson GE, Moller A, Blomstrand C. Consequences of mild stroke in persons <75 years -- a 1-year follow-up. *Cerebrovasc Dis* 2003;16(4):383-8. doi: 10.1159/000072561

5. Cumming TB, Marshall RS, Lazar RM. Stroke, cognitive deficits, and rehabilitation: still an incomplete picture. *Int J Stroke* 2013;8(1):38-45. doi: 10.1111/j.1747-4949.2012.00972.x

6. Levasseur M, Carrier A. Integrating health literacy into occupational therapy: Findings from a scoping review. *Scand J Occup Ther* 2012;19(4):305-14. doi: 10.3109/11038128.2011.588724

7. Mackey LM, Doody C, Werner EL, et al. Self-Management Skills in Chronic Disease Management: What Role Does Health Literacy Have? *Med Decis Making* 2016;36(6):741-59. doi: 10.1177/0272989X16638330 [published Online First: 2016/04/08]

8. Kirkevold M. The unfolding illness trajectory of stroke. *Disabil Rehabil* 2002;24(17):887-98. doi: 10.1080/09638280210142239

9. Tistad M, Ytterberg C, Tham K, et al. Poor concurrence between disabilities as described by patients and established assessment tools three months after stroke: a mixed methods approach. *J Neurol Sci* 2012;313(1-2):160-6. doi: 10.1016/j.jns.2011.08.038 [published Online First: 2011/09/23]

10. Ekman I, Swedberg K, Taft C, et al. Person-centered care—Ready for prime time. *Eur J Cardiovasc Nurs* 2011;10(4):248-51.

11. Philp I, Brainin M, Walker MF, et al. Development of a poststroke checklist to standardize follow-up care for stroke survivors. *J Stroke Cerebrovasc Dis* 2013;22(7):e173-80. doi: 10.1016/j.jstrokecerebrovasdis.2012.10.016

For peer review only - http://bmjopen.bmj.com/site/about/guidelines.xhtml

12. Ward AB, Chen C, Norrving B, et al. Evaluation of the Post Stroke Checklist: a pilot study in the United Kingdom and Singapore. *Int J Stroke* 2014;9 Suppl A100(A100):76-84. doi: 10.1111/ijs.12291
13. Iosa M, Lupo A, Morone G, et al. Post Soft Care: Italian implementation of a post-stroke checklist software for primary care and identification of unmet needs in community-dwelling patients. *Neurol Sci* 2017;39(1):135-39. doi: 10.1007/s10072-017-3140-1
14. Hotter B, Padberg I, Liebenau A, et al. Identifying unmet needs in long-term stroke care using in-depth assessment and the Post-Stroke Checklist – The Managing Aftercare for Stroke (MAS-I) study. *European Stroke Journal* 2018;3(3):237-45. doi: 10.1177/2396987318771174
15. Krueger RA. Focus groups: A practical guide for applied research. 5th ed. London: Sage publications 2014.
16. Ivanoff SD, Hultberg J. Understanding the multiple realities of everyday life: basic assumptions in focus-group methodology. *Scand J Occup Ther* 2006;13(2):125-32.
17. Craig P, Dieppe P, Macintyre S, et al. Developing and evaluating complex interventions: the new Medical Research Council guidance. *BMJ* 2008;337:a1655. doi: 10.1136/bmj.a1655
18. Beaton DE, Bombardier C, Guillemin F, et al. Guidelines for the process of cross-cultural adaptation of self-report measures. *Spine (Phila Pa 1976)* 2000;25(24):3186-91. [published Online First: 2000/12/22]
19. Tong A, Sainsbury P, Craig J. Consolidated criteria for reporting qualitative research (COREQ): a 32-item checklist for interviews and focus groups. *Int J Qual Health Care* 2007;19(6)
20. Malterud K, Siersma VD, Guassora AD. Sample Size in Qualitative Interview Studies: Guided by Information Power. *Qual Health Res* 2015 doi: 10.1177/1049732315617444 [published Online First: 2015/11/29]
21. Paul SL, Srikanth VK, Thrift AG. The large and growing burden of stroke. *Curr Drug Targets* 2007;8(7):786-93. [published Online First: 2007/07/17]
22. Stevens E, Emmett E, Wang DY, et al. The Burden Of Stroke In Europe Report London: SAFE Stroke Alliance For Europe 2017.
23. Norrving B, Barrick J, Davalos A, et al. Action Plan for Stroke in Europe 2018–2030. *European Stroke Journal*;0(0):2396987318808719. doi: 10.1177/2396987318808719

For peer review only - http://bmjopen.bmj.com/site/about/guidelines.xhtml

24. Shannon RL, Forster A, Hawkins RJ. A qualitative exploration of self-reported unmet need one year after stroke. *Disabil Rehabil* 2016;38(20):2000-7. doi: 10.3109/09638288.2015.1107784

25. Williams MV, Davis T, Parker RM, et al. The role of health literacy in patient-physician communication. *Fam Med* 2002;34(5):383-9.

26. Silverman J, Kurtz S, Draper J. Skills for communicating with patients. 3rd ed. London: CRC Press 2016.

For peer review only

For peer review only - http://bmjopen.bmj.com/site/about/guidelines.xhtml

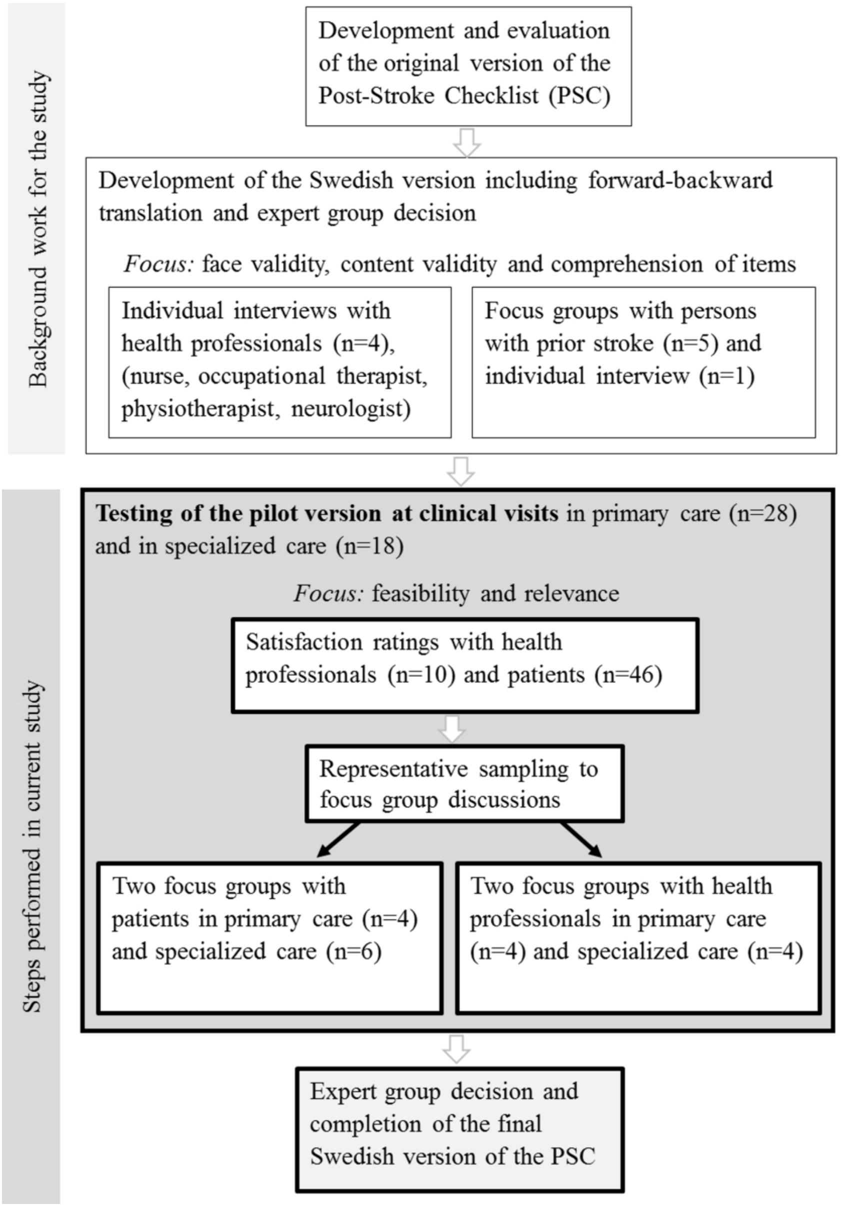

Figure 1. The steps included in the validation and cross-cultural adaptation of the Swedish version of the PSC including background work and current study.

199x267mm (300 x 300 DPI)

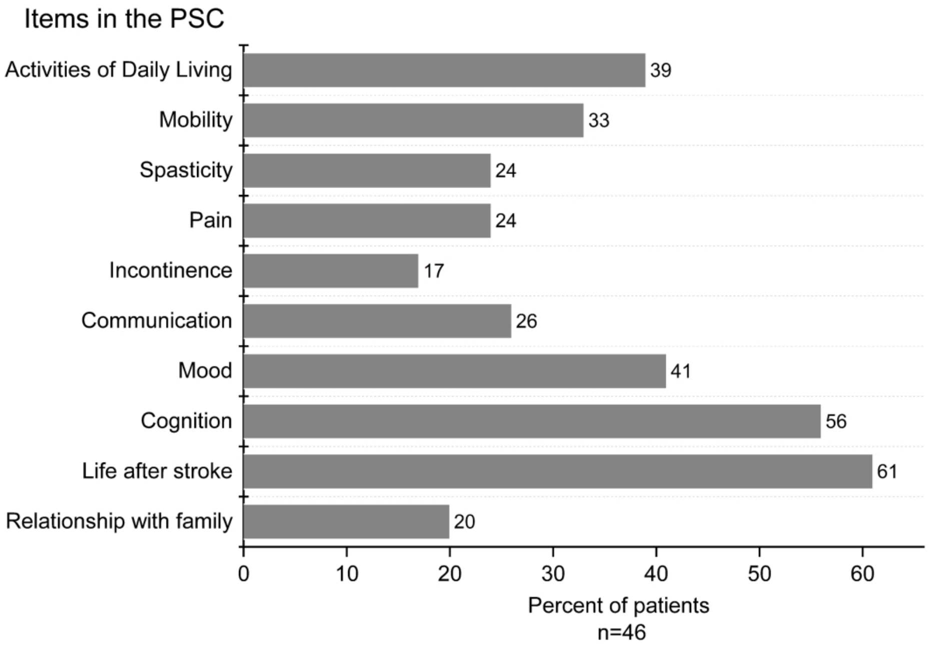

Figure 2. Percentages of patients with identified problems in each Post-Stroke Checklist item (n=46).

229x160mm (300 x 300 DPI)

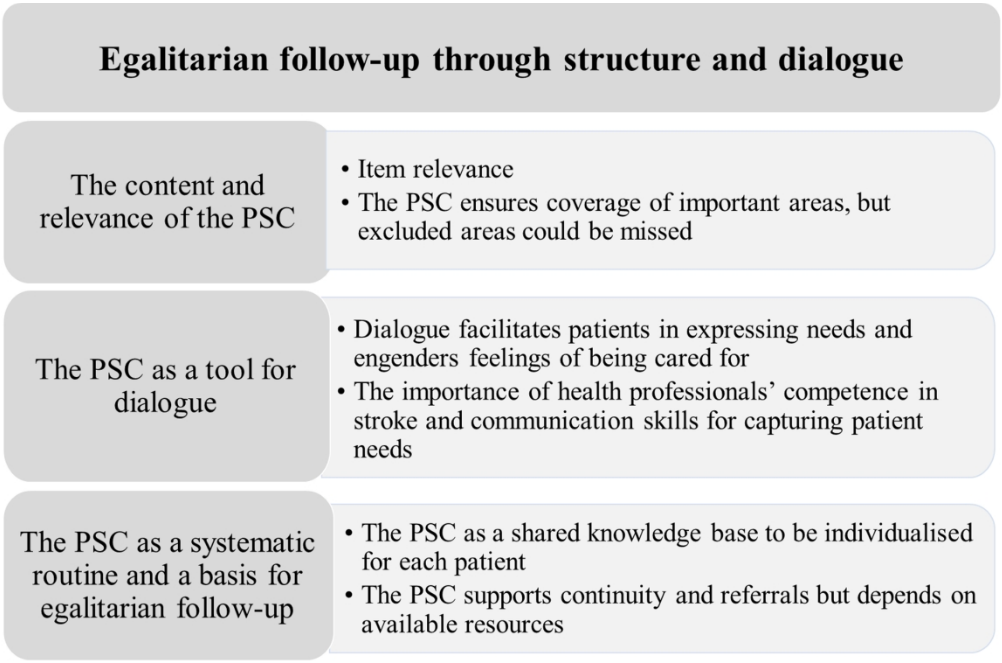

Figure 3. Themes and subthemes derived from the focusgroup discussions with patients and health professionals regarding experiences of using the Post-Stroke Checklist.

170x112mm (300 x 300 DPI)

Appendix 2 - COREQ 32-item checklist

**No. Item**	**Guide questions/description**	**Comments**
**Domain 1: Research team and reflexivity**		
Personal characteristics		
1. Interviewer/facilitator	Which author/s conducted the interview?	EK (p.6)
2. Credentials	What were the researcher's credentials? *e.g., PhD, MD*	3 Occupational Therapists (1 associate professor, 1 PhD, and 1 MS, PhD student) 1 Medical doctor, (Professor)
3. Occupation	What was their occupation at the time of the study?	Researchers
4. Gender	Was the researcher male or female?	4 female (p. 6-7)
5. Experience and training	What experience or training did the researchers have?	> 20 years in stroke rehab, Qualitative research (p. 7)
Relationship with participants		

For peer review only

6. Relationship established	Was a relationship established prior to study commencement?	The moderator had met some of the health professionals in work related situations.
7. Participant knowledge of the interviewer	What did the participants know about the researcher? *e.g., personal goals, reasons for doing the research*	Broad outlines given
8. Interviewer characteristics	What characteristics were reported about the interviewer/facilitator? *E.g., bias, assumptions, reasons and interests in the research topic*	(p. 5)
**Domain 2: study design**		
Theoretical framework		
9. Methodological orientation and Theory	What methodological orientation was stated to underpin the study? *e.g. grounded theory, discourse analysis, ethnography, phenomenology, content analysis*	Focus group according to Kreuger, underpinned by a methodology based on Social constructivism (p. 4)
Participant selection		

For peer review only

10. Sampling	How were participants selected? *e.g. purposive, convenience, consecutive, snowball*	Purposive (p. 5)
11. Method of approach	How were participants approached? e.g. *face-to-face, telephone, mail, email*	Face to face and by telephone (p. 5)
12. Sample size	How many participants were in the study?	In total, 18 (patients/HPs) participated in the focus groups (see table 2, p 7)
13. Non-participation	How many people refused to participate or dropped out? Reasons?	One dropped out because of a scheduled medical examination (p. 9)
Setting		
14. Setting of data collection	Where was the data collected? *e.g. home, clinic, workplace*	Clinical setting (p. 5)
15. Presence of non-participants	Was anyone else present besides the participants and researchers?	No (p. 6)
16. Description of sample	What are the important characteristics of the sample? *e.g. demographic data, date*	People with stroke in a late phase of stroke recovery (p. 7, table 2)
Data collection		

For peer review only

17. Interview guide	Were questions, prompts, guides provided by the authors? Was it pilot tested?	A interview guide was provided (p.6). A pilot testing was conducted (p. 5).
18. Repeat interviews	Were repeat interviews carried out?	No, each group met once (p. 6)
19. Audio/visual recording	Did the researchers use audio or visual recording to collect the data?	Audio (p. 6)
20. Field notes	Were field notes made during and/or after the interview or focus group?	Yes
21. Duration	What was the duration of the interviews or focus group?	Approximately 1.5 hours (p. 6)
22 Data saturation	Was data saturation discussed?	Yes (p. 6)
23. Transcripts returned	Were transcripts returned to participants for comment and/or correction?	No
**Domain 3: analysis and findings**		
Data analysis		
24. Number of data coders	How many data coders coded the data?	Two (EK and GC) (p. 6)
25. Description of the coding tree	Did authors provide a description of the coding tree?	Yes.
26. Derivation of themes	Were themes identified in advance or derived from the data?	Derived from the data (p. 6)

For peer review only

27. Software	What software, if applicable, was used to manage the data?	Nvivo (p. 6)
28. Participant checking	Did participants provide feedback on the findings?	No, not specifically, but a general presentation (p. 5)
Reporting		
29. Quotations presented	Were participant quotations presented to illustrate the themes/findings? Was each quotation identified? *e.g. participant number*	Yes (Results)
30. Data and findings consistent	Was there consistency between the data presented and the findings?	Yes (Results and Discussion)
31. Clarity of major themes	Were major themes clearly presented in the findings?	Yes (Figure 3 and results)
32. Clarity of minor themes	Is there a description of diverse cases or discussion of minor themes?	Yes (Results and Discussion)

For peer review only

BMJ Open

Experiences using the Post-Stroke Checklist in Sweden with a focus on feasibility and relevance: a mixed-method design

Journal:	*BMJ Open*
Manuscript ID	bmjopen-2018-028218.R1
Article Type:	Research
Date Submitted by the Author:	28-Jan-2019
Complete List of Authors:	Kjörk, Emma K; University of Gothenburg, Institute of Neuroscience and Physiology, Department of Clinical Neuroscience<br>Carlsson, Gunnel; University of Gothenburg, Institute of Neuroscience and Physiology, Department of Clinical Neuroscience<br>Sunnerhagen, Katharina; University of Gothenburg, Institute of Neuroscience and Physiology, Department of Clinical Neuroscience<br>Lundgren-Nilsson, Asa; Gothenburg University, Institute of Neuroscience and Physiology, Department och Clinical Neuroscience
**Primary Subject Heading**:	Cardiovascular medicine
Secondary Subject Heading:	Evidence based practice, Health services research, Rehabilitation medicine
Keywords:	long-term care, Feasibility, Focus groups, Follow-up, Standard of Care, Stroke < NEUROLOGY

SCHOLARONE™
Manuscripts

Experiences using the Post-Stroke Checklist in Sweden with a focus on feasibility and relevance: a mixed-method design

Emma K Kjörk* OT, MS[1, 2], Gunnel Carlsson OT, PhD[1], Katharina S Sunnerhagen MD, PhD, Prof[1] and Åsa Lundgren-Nilsson OT, PhD[1]

[1]Institute of Neuroscience and Physiology, Dep of Clinical Neuroscience, Sahlgrenska Academy at University of Gothenburg, Gothenburg, Sweden
[2]Department of Occupational Therapy and Physiotherapy, Sahlgrenska University Hospital, Gothenburg, Sweden

* Corresponding author:
Emma K Kjörk
Email address: emma.kjork@neuro.gu.se
Telephone: +46313422803
Postal address:
Institute of Neuroscience and Physiology
Dep of Clinical Neuroscience, Rehabilitation medicine
Per Dubbsgatan 14, fl. 3, 413 45 Gothenburg, Sweden

Word count Abstract: 300 words
Word count Main manuscript: 3991 words
Number of tables: 3
Number of figures: 3

For peer review only - http://bmjopen.bmj.com/site/about/guidelines.xhtml

Experiences using the Post-Stroke Checklist in Sweden with a focus on feasibility and relevance: a mixed-method design

ABSTRACT

Objective The wide range of outcomes after stroke emphasises the need for comprehensive long-term follow-up. The aim was to evaluate how people with stroke and health professionals (HPs') perceive the use of the Post-Stroke Checklist (PSC), with a focus on feasibility and relevance.

Design An exploratory design with a mix of qualitative and quantitative methods.

Setting Outpatient care at a university hospital and primary care centres in western Sweden.

Participants Forty-six consecutive patients (median age, 70; range, 41–85; 13 women) and ten health professionals (median age 46; range, 35-63; 7 women).

Results Most patients (87%) had one or more problems identified by the PSC. The most common problem areas were life after stroke (61%), cognition (56%), mood (41%), and activities of daily living (39%). Three organisational themes emerged from the focus group discussions. The perception of *the content and relevance of* the PSC was that common post-stroke problems were covered but that unmet needs still could be missed. Identifying needs was facilitated when using the *PSC as a tool for dialogue.* The dialogue between the patient and HP as well as HPs stroke expertise was perceived as important. The PSC was seen as *a systematic routine and a base for egalitarian follow-up*, but participants stressed consideration given to each individual. Addressing identified needs and meeting patient expectations were described as challenging given available healthcare services.

Conclusions The PSC is a feasible and relevant tool to support egalitarian follow-up and identify patients who could benefit from targeted post-stroke interventions. Stroke expertise, room for dialogue, and caring for identified needs emerged as important issues to consider when using the PSC. Nutrition, sexuality and fatigue were areas mentioned that might need to be addressed within the discussions. The PSC can facilitate patients in expressing their needs, enhancing their ability to participate in decision-making.

For peer review only - http://bmjopen.bmj.com/site/about/guidelines.xhtml

Keywords Stroke, Rehabilitation, Standard of care, Feasibility, Focus groups, Follow-up

Strengths and limitations (bullet points)

- A strength of this study is having patients as partners throughout the project, from the translation process to participation in the focus groups.

- The mix of methods made it possible to explore the feasibility of the PSC from different perspectives and at different levels compared to previous research.

- A limitation is the lack of data collected from people with severe stroke, although this lack is typical of this naturalistic design and representative for the Swedish stroke population.

- A study strength is the heterogeneous population from different outpatient settings, including a range of ages, stroke characteristics, and education levels, and health professionals with a range of experience in stroke care.

- The study was conducted in a Swedish context, and the transferability of the findings in other cultural contexts is not known.

For peer review only

For peer review only - http://bmjopen.bmj.com/site/about/guidelines.xhtml

INTRODUCTION

Post-stroke impairments often have long-term negative consequences for social relationships, dependence in daily life, and quality of life.[1] Perceived unfulfilled rehabilitation needs and changes in functioning during the first year after stroke[2 3] indicate the need for systematic long-term follow-up. Furthermore, a subtle decline in cognition as well as emotional problems[4 5] can easily be overlooked, leading to difficulty in accessing healthcare services.[6 7]

The adaptation process after stroke is long. In later phases, the focus changes from the stroke itself to more familiar aspects of daily life,[8] which may not be sufficiently targeted in current practice. Concordance is poor between perceived problems and problems detected by standardised assessments. Accordingly, dialogue should complement assessments to ensure that health services are based on patient needs,[9] in line with a person-centred approach.[10]

The Post-Stroke Checklist (PSC)[11] was developed to identify long-term care needs and facilitate referrals.[11 12] Although the PSC has been found to be feasible and useful,[12-14] knowledge is lacking about the perspectives of patients and health professionals (HPs) regarding its use in the Swedish health care context. The aim of this study was to evaluate how people with stroke and HPs perceive the use of the PSC, with a focus on feasibility and relevance.

METHODS

Study design

The study has an exploratory design. To capture the feasibility of using the PSC, we combined it with a satisfaction questionnaire and focus group discussions,[15 16] in line with guidelines for complex interventions.[17] This study is part of a validation and cultural adaptation process[18] of the PSC in Sweden (figure 1). By combining data collection methods, we expected to gain a deeper understanding of using the PSC as a tool to structure follow-up. The underpinning methodology in the focus groups is based on social constructivism.[16] Consolidated criteria for reporting qualitative research (i.e., COREQ guidelines)[19] were followed for reporting qualitative data.

[Insert figure 1 near here]

For peer review only - http://bmjopen.bmj.com/site/about/guidelines.xhtml

Patient and public involvement

This study explores how people with stroke experience the PSC. People from the Swedish Stroke Association (patient association) were involved in the translation process, the pilot testing of the interview guide, and focus group discussions and have been given a presentation of preliminary results.

Participants

Participants were consecutively recruited while at a clinical visit in primary care or stroke specialised outpatient care at a university hospital, February 2015 to October 2015. The inclusion criterion was having had a stroke, regardless of the time of onset. The number of patients included was in accordance with a previous study[12] and principles for cross-cultural adaptation suggesting approximately 40.[18] Patients were excluded if cognitive impairment or insufficient knowledge of Swedish would have made the response to the PSC items unreliable. HPs from different clinics were invited to participate and selected to represent different professions. A purposive sampling was used with the attempt to achieve heterogeneity and homogeneity in the focus groups.[15 16] Written informed consent was obtained from all participants, and the regional ethical review board in Gothenburg approved the study (no. 521-14).

Data collection

The PSC[11] has 11 items and is intended to identify post-stroke problems. It was developed by a multi-professional group of stroke experts, according to a Delphi process. Problem areas were chosen for having the greatest impact on patient quality of life that could be addressed with evidence-based interventions. The PSC includes secondary prevention, activities of daily living (ADLs), mobility, spasticity, pain, incontinence, communication, mood, cognition, life after stroke, and relationship with family. A response scale includes 'yes' and 'no' and recommended referrals adjacent to each problem area.[11]

Data collection was conducted in outpatient clinical facilities in two steps. For step 1, the HP administered the PSC to patients at a regular clinical visit (proxy responses were allowed). No additional training was given beyond the general instructions on the PSC. Participants were asked to reflect on the usefulness of the PSC as the basis for focus group discussions. Patients and HPs assessed satisfaction with the PSC after each visit through a satisfaction questionnaire with questions analogous to those used in a previous study.[12] The answers were rated on a Likert scale of 1–5, where 1 indicated not satisfied and 5 completely

For peer review only - http://bmjopen.bmj.com/site/about/guidelines.xhtml

satisfied. To ensure anonymity, patient responses were collected in an envelope. Demographic data (time since index stroke, age, sex), time to administration of the PSC, and HP profession were registered. In addition, if any referrals were made it was registered as yes/no for each patient without specification of what kind of referrals or standardised "actions" to be taken. Of the patients who gave informed consent, additional patient characteristics (such as type of stroke, aphasia, ADL dependency) were collected retrospectively from their medical records.

For step 2, the staff invited all participants during the follow-up to join in a focus group discussion. A set time of approximately 1-2 months was given between the visit and the focus group discussions. The first author (EK) telephoned participants willing to participate and sent them the study information letter, time for appointment, and a copy of the PSC. In total, four focus groups were conducted using a question guide.[15] Each focus group met once for approximately 1.5 hours. The meeting was recorded and transcribed verbatim. Initially in the focus groups, the importance of bringing up different opinions was emphasised. At the end, an oral overview was presented to ensure that participant contributions were as they intended.

Data analysis

Data gathered from PSC items, questionnaires, and demographic information were analysed using descriptive statistics with Statistical Package for the Social Sciences version 24. Data gathered from focus groups were analysed following Kreuger.[15] The aim of the analysis process was to describe the participants' perceptions and experiences based on the aim of the study.

Immediately after each focus group discussion, a written summary was created. The transcripts were coded using the computer software NVivo. First, the transcripts were read in their entirety to allow familiarity with the content as a whole, and discussions relevant to the aim were identified. Second, transcripts were systematised into categories based on similarities and differences in the discussions. Third, a descriptive summary was made for each category, adhering as closely as possible to the content of the raw data. Finally, these summaries in combination with selected quotes served as the basis for the interpretation and presented a deeper insight into the findings. In the analysis process, identified patterns were compared and contrasted across all four groups, resulting in an overarching thematic structure (for examples of the coding tree and themes see table 1). Quotations that showed the ongoing discussions[16] were selected to illuminate the results. Based on sampling strategies[20] and when similar discussions recurred in all groups[15], data gathering stopped.

For peer review only - http://bmjopen.bmj.com/site/about/guidelines.xhtml

The first author (PhD student, OT, woman) was the moderator and performed most of the analysis. Multiple coding, continuous interpretation of data, and discussion of the emerging themes were completed together with the second author (PhD, OT, woman) to ensure accuracy of the analysis. The first author had knowledge about the study topic and the second author in qualitative methods. Both have conducted interviews previously. The third author (PhD, MD, woman) and last author (PhD, OT, woman) contributed with knowledge concerning revising and refining the themes. All authors have at least 20 years of experience in stroke rehabilitation.

Table 1. Examples illustrating the coding tree.

Quote	Code	Subtheme
P4:"It has a lot to do with the competence of the person who's asking the questions so they can do the thinking to squeeze it all in" (Group 1 Patients)	The professionals' expertise and reasoning	The importance of HPs' with stroke expertise and communication skills for capturing patient needs
P2: "So, I think it has just been positive, and it is also done so quickly". P1: "That's also a positive". P2: "Yeah, it's fast, but you can also develop it as much as you want. But asking the questions doesn't take too long". Moderator: "Is it quick?" P1: "Yes. It also depends on what answers you get". P2: "Yes". (Group 4. Health professionals)	The administration of the PSC can be adapted, quickly done or more in-depth	The PSC supports continuity and referrals but depends on available resources

RESULTS

Study group

The PSC was used in connection with a clinical visit in 46 patients. All patients lived in their homes. Most of them (65%) had experienced a stroke within 3 months at enrolment. The median time for hospitalisation was 8 days. Stroke severity at stroke onset was mild, with a

For peer review only - http://bmjopen.bmj.com/site/about/guidelines.xhtml

median of 2 according to National Institutes of Health Stroke Scale. Table 2 shows the characteristics of the participants and the focus groups.

Table 2. Characteristics of patients and health professionals in the clinical outpatient visits and the focus group discussions. Data are presented as number of persons (n) or median and range.

**Patients**	Clinical visit (n=46)	Focus group 1 (n=4)	Focus group 2 (n=6)
Primary care, rural		x	
Specialized care, urban			x
Age at inclusion	70 (41-85)	71 (58-78)	74 (45-76)
Sex, male	33, 72%	4	5
Education			
Mandatory	20	1	4
High School	13	1	1
University	8	2	1
Months since stroke	3 (1-84)	20 (3-84)	3 (1-6)
Working at stroke onset (yes)	13	2	1
Length of hospitalization, (days)	8 (2-120)	11 (5-82)	8 (4-11)
History of stroke (yes)	9	1	3
Stroke characteristics			
Ischemic/Haemorrhagic	36/5	4/0	4/2
Right/ left/ posterior/ bilateral	19/16/5/2	3/1/0/0	3/2/1/0
NIHSS	2 (0-16)	4 (3-10)	2 (1-6)
Aphasia (yes)	9	0	1
Neglect (yes)	4	1	0
At discharge			
ADL independency (yes)	34	3	6
Wheel-chair use (yes)	4	1	0
**Health Professionals**	Clinical visit (n=10)	Focus group 3 (n=4)	Focus group 4 (n=4)
Age	46 (35-63)	43 (37-46)	46 (35-55)
Primary care, rural			x
Specialized care, urban		x	
Sex, male	3, 30%	0	1
Nurse/ OT/ Physician	4/1/5	3/0/1	0/1/3
Stroke experience (years)			
≤5/ 5-10/ 10	2/2/6	0/1/3	2/1/1

Abbreviations: NIHSS=National Institutes of Health Stroke Scale, ADL= Activities of Daily Living, OT= Occupational Therapist.

(Missing data from medical records (n=4).

For peer review only - http://bmjopen.bmj.com/site/about/guidelines.xhtml

Feasibility of the PSC

Forty patients (87%) had one or more problems identified by the PSC (figure 2). 'Life after stroke' was most common (61%), followed by cognition (56%) and mood changes (41%). A median of four problem areas per patient (range, 0–9; interquartile range [IQR], 1–5) was identified; the median in specialised care was 3 (IQR, 1–5), and in primary care, it was 4 (IQR, 1–5). Only six (13%) patients reported no problems. Most patients (70%) acknowledged having received information about secondary prevention. Referrals were registered in eight cases, slightly more often in primary care (n=6) than in specialised care (n=2). The time taken to administer the PSC was ≤15 minutes for 52%, ≤30 minutes for 43%, and ≥45 minutes for 5%.

[Insert figure 2 near here]

Four focus groups were conducted, and their characteristics are shown in table 2. One woman dropped out because of a scheduled medical examination. The focus group discussions revealed that the PSC structure in combination with room for dialogue could support egalitarian follow-up and identification of needs. A main theme and three organisational themes emerged in these discussions (figure 3).

[Insert figure 3 near here]

The content and relevance of the PSC

Item relevance

The items included in the PSC were considered relevant to all groups. Because stroke affects persons differently, participants found it valuable that the PSC covers a broad spectrum of problems, although not all problems are relevant to every person with stroke.

The PSC ensures coverage of important areas, but excluded areas could be missed

Both patients and HPs stated that some issues might be overlooked if not specifically stated in the PSC; such as nutrition, sexuality, vision, irritability and driving. The HPs discussed the appropriate amount of problem areas in the PSC. They wanted more areas yet preferred the checklist to be short and complemented by profession-specific assessments when needed. Some participants appreciated the recurring phrase "since your stroke", but others preferred to hear/say it once at the beginning of the visit. Patients perceived the PSC as easy to

For peer review only - http://bmjopen.bmj.com/site/about/guidelines.xhtml

understand, while HPs expressed a concern about misunderstandings, especially for the items ‘secondary prevention’ and ‘spasticity’.

The PSC as a tool for dialogue

Patients and HPs both emphasised the need for dialogue to create mutual understanding. HPs described that knowledge and experience affected their ability to detect problems, while patients described differences in their ability to communicate problems.

Dialogue facilitates patients in expressing needs and engenders feelings of being cared for

Patients said that the PSC questions facilitated dialogue, leading to a greater likelihood that important areas would be elucidated and discussed. Memory problems, lack of initiative, fatigue, or being less talkative were mentioned as barriers to dialogue that the PSC could address. The PSC gave clear direction for the structure of the dialogue and accordingly facilitating identification of problems. Nevertheless, using the PSC in combination with dialogue was seen as important. Patients stated that they might need time for consideration before answering the PSC questions, time that was often not given within the limits of the visits.

Generally, participants thought patients should have the opportunity to talk to a professional about stroke-related concerns and stated that the PSC could facilitate this exchange. Patients expressed that the PSC covers areas centring on them as a person, which made them feel cared for. The understanding of the HPs was that relatives often complemented information concerning problems that patients might neglect or forget to mention:

> *P4: “I think there is a great deal of importance to how much you are affected by the stroke. The more you are affected, the harder it is to think about the different facets of it”* (the areas in the PSC). (Strongly agreed upon)
> (Group 1. Patients)

The importance of HPs’ with stroke expertise and communication skills for capturing patient needs

To ensure that problems would be fully addressed, a professional’s competence in stroke was seen as key by the participants. HPs feared that lack of HP’s with stroke expertise might lead

For peer review only - http://bmjopen.bmj.com/site/about/guidelines.xhtml

to problems going unrecognised. The PSC was seen as an asset as well as a barrier to dialogue. If too much focus was placed on posing the questions, participants experienced a decreased interaction. Sensitivity from the HPs and use of additional questions was seen as essential (e.g. work issues, within the item 'life after stroke'):

> *P7: "No, there's only benefits, but it depends on how you use it, and if the staff think it is meaningful, so it's not just checked off. Rather that you have the opportunity to cover the things that each point is actually about".*
> *P8: "Yeah, all the stuff that's crazy [difficulties after the stroke], follow up that stuff."*
> *Moderator: "That you have time to follow up what is included in the point? Can you elaborate on that?"*
> *P7: "Yes, it's the topic this question is about, that you can elaborate on if you want to (...) the person with the checklist shouldn't be bound to it 100% and slavishly follow it, but understand signals from the patient and talk more broadly and connect it to the other things that depend upon it". (agreement)*
> *P9: "Absolutely".*
> *P7: "Otherwise, it just becomes mechanical; you can't be just like a computer asking questions".*
> (Group 2. Patients)

There were conflicting opinions about how to apply the questions in the PSC. Experienced HPs preferred to use it as a supplement for memory within a free dialogue. In contrast, inexperienced HPs perceived the specific questions as good to assume and a basis for leading into other related concerns (e.g. fatigue):

> *P5: "It's more about if you think that you should use standardised things for everyone, even for primary healthcare/outpatient care, I don't think that really works".*
> *P6: "Exactly, that's the question, should you use the checklist just as it is, or should you use it for your own part and remember. That's the thing, because you can then approach each patient differently and get it all. But asking the exact same questions for each patient, I agree, that's really hard to do".*
> (Group 3. HPs)

For peer review only - http://bmjopen.bmj.com/site/about/guidelines.xhtml

PSC as a systematic routine and a basis for egalitarian follow-up

The PSC as a shared knowledge base to be individualised for each patient

The PSC was considered to increase knowledge about stroke and secure an egalitarian follow-up, especially for inexperienced HPs and patients with limited ability to express their needs. Even when the PSC was used, lack of HPs with stroke expertise and limited knowledge about opportunities for referrals were perceived as an obstacle to egalitarian follow-up. One suggestion was to add local referral opportunities and access to scientific references in conjunction with the PSC. Factors such as comorbidities and time since stroke must be taken into account because they could affect responses to PSC items.

The PSC supports continuity and referrals but depends on available resources

Participants addressed the need for regular follow-ups and considered the PSC to be a useful tool and basis for referrals. The use of the PSC was seen as a rapid way to cover the problem areas if only the questions were used, but when supplementary questions were needed, the time need also increased. One concern, especially among the physicians, was the time taken to administer the PSC in addition to their ordinary routines. HPs emphasised that the use of the PSC should be beneficial for the patients and in accordance with time limits and referral opportunities. Some considered it a bit rigid to go through all items if the patient experienced no problems, although it was observed that it could be completed quickly.

To enable preparation beforehand and make visits more time-efficient, a patient version of the PSC was proposed. Participants strongly emphasised that problems identified by the PSC should lead to appropriate intervention and not only an evaluation of current status:

> *P11: "The risk is you might get a false sense of security though. So, someone has asked the question, and I have answered "yes" to this question; so I then expect something to happen".*
>
> (agreement from the others)
>
> *P11: "It's like, that's what decides the quality of what happens with the measures (…). It should end with me knowing how this information is taken and handled, what happens now. Not just that you do it and then that's great. (mumbles) Is it like statistics or what?"*
>
> (Group 1. Patients)

For peer review only - http://bmjopen.bmj.com/site/about/guidelines.xhtml

Lack of opportunities for interventions as well as knowledge gaps were expressed by HPs as difficulties in meeting these expectations. A specific dimension of this problem was mentioned as leading to a risk of avoidance of discussing certain items.

Table 3. Evaluation of the use of Post-Stroke Checklist (PSC) based on satisfaction ratings (Likert 1-5) by patients and health professionals.

Satisfaction with:	Patients (n=46) median (IQR)	Health professionals (n=10) median (IQR)
Overall assessment where PSC was used	5 (4-5)	4 (3-4)
Identification of needs	5 (4-5)	-
Identification of need (for each patient)	-	3 (3-4)
Confidence in receiving support	5 (4-5)	-
Guidance for referrals and treatment	-	3 (2-4)

Abbreviations: IQR= interquartile range

By combining the results derived by different methods, additional aspects of the analysis can be demonstrated. The patients evaluated the satisfaction with the PSC as high (table 3). In addition, the focus group analyses gave insights into a wide range of factors that affect satisfaction, and its feasibility was exemplified by the importance of dialogue (figure 3). HP satisfaction with the PSC varied among patients (table 3). Participants perceived it as important to adapt the use of the PSC to individual aspects. Some differences regarding individual prerequisites are displayed in table 2, and others are mentioned in the qualitative analyses. HP stroke experience varied, especially in the primary care settings (table 2). In the focus group discussions, HPs with stroke expertise was perceived as important if subtle problems were to be properly recognised.

DISCUSSION

The PSC is a relevant and feasible tool to identify patients who can benefit from targeted interventions, as noted by people with stroke and HPs. The original purpose of the PSC was to be an easy-to-use tool to detect post-stroke problems, as well as a support for guiding referrals.[11] This study brings out an awareness about how follow-up through the PSC could be enhanced by user perceptions and suggested strategies. This knowledge could add important insights when implementing the PSC in line with the World Stroke Organization recommendations. The focus group discussions raised issues concerning prerequisites when

For peer review only - http://bmjopen.bmj.com/site/about/guidelines.xhtml

using the PSC. These include HP with stroke expertise, room for dialogue, and how the identified needs were addressed.

The wide range of post-stroke problems identified in the present study demonstrated the relevance of the PSC, with a median of four problems per patient. Of note, reported problems on specific PSC items differ considerably; 'life after stroke', cognition, and continence vary when comparing among countries.[12-14] Comparison should be made with caution since the groups studied differs with respect to e.g. case-mix, sampling strategies and inclusion criteria in the studies. Further, based on issues raised in the focus group discussions, likely causes of these discrepancies in reported problems could be HP stroke expertise, opportunity for a dialogue, and time limits on the administration of the PSC. Comorbidities also could affect responses to the PSC items due to respondents not being able to consider whether the problems are stroke related or not. Nevertheless, the wide range of identified problems alongside participant perceptions in this study stresses the relevance of using the PSC in clinical practice. The long-term consequences after stroke emphasise the need for a comprehensive long-term follow-up with a multi-domain approach.[1 3 21-23]

The present study provides a deeper understanding of how the PSC structure could support patients in expressing their needs. Problems with communication and comprehension are common after stroke,[5] which influences decision-making during follow-up. Participation in decision-making requires health literacy, i.e., the ability to understand health information and a capacity to argue for one's needs in relation to appropriate interventions.[7] In this study, despite perceptions that the PSC questions were easy to understand, dialogue was found to be crucial. Participants raised concerns about problem areas that could be missed depending on how the PSC was used. Results from using the PSC in the UK and Singapore[12] indicate that several problem areas could be indirectly identified. Awareness of the complexity of need identification underscores the role of the HP when using the PSC. Even if no unmet need is reported, a person can still identify as living with residual impairments and perceived problems in engaging in activities.[24] Participants expressed that identification of needs could be enhanced if more time were allowed for consideration of these needs and for additional questions; another help would be the opportunity to fill in the PSC beforehand. Current findings stress HP with stroke expertise and the need to make space for dialogue when administering the PSC to support needs identification and decision-making.

The results from the present study highlight the dialogue between the patient and HP, which is central in health care.[9 10 25] Experienced HPs argued that they could cover most topics using open-ended questions. In contrast, others emphasised the value of articulating the

For peer review only - http://bmjopen.bmj.com/site/about/guidelines.xhtml

PSC questions literally. To enable investigation of specific areas, closed questions can be important[26] and facilitate situations involving patients with communication difficulties. However, the PSC instructions do not hinder its use in a looser way as long as all areas are captured. The result shows that patients can benefit from a clear structure when the PSC is used. Participants in all focus groups agreed on the benefits of going through the areas in the PSC in a way that ensures identification of unmet needs. In addition, using the PSC in combination with dialogue supports the patients' capacity to communicate their needs. A narrative communication, along with signs of problems, gives the HP a foundation for planning care together with the patient and creates conditions for patients to make appropriate health decisions.[10]

The PSC can improve clinical pathways in health care by its structure and guidance for further referrals. Creating a plan to take care of identified needs and locally adapted pathways to support access to appropriate interventions, was noted in the focus group discussions as essential.

Strengths and limitations

A strength of this study is that patients were partners throughout the project, from the translation process to participation in the focus group discussions. In addition, the mix of methods made it possible to explore the feasibility of the PSC at different levels compared to previous research. Few persons in this population had lived with their stroke for a long time, and only one of them had a severe stroke, which might have affected which problems were identified (e.g. spasticity). This naturalistic design in an ordinary outpatient context, however, is representative of the Swedish population, where the majority have mild stroke.[27] The time (1-2 months) between the follow-up visit where the PSC was used and the focus group discussion could be a risk for recall bias. However, the focus group methodology enabled exploring a range of opinions of people across groups, and together, the participants contributed to rich discussions. Although the attempt was to obtain heterogeneity and homogeneity in the focus groups, the majority of HPs were women and the majority of the patients were male. Because the purposive sampling of HPs were made based on health care facilities already chosen and the defined time limit between the visit and the focus groups, the sex distribution were out of our influence. However, heterogeneity was achieved with respect to different outpatient settings, patients having a range of ages, stroke characteristics, and education levels, and HPs with a range of professional roles and experience in stroke. To strengthen the transferability of the findings, a comprehensive description of the study

For peer review only - http://bmjopen.bmj.com/site/about/guidelines.xhtml

context, participant characteristics, data collection, and analysis process are included in the methods section. Nevertheless, there are limitations regarding the transferability of the findings outside of the Swedish healthcare context. To ensure the feasibility of using the PSC in another context, a cross-cultural validation is needed. However, because the World Stroke Organization recommends using the PSC globally, these results contribute to a deeper understanding of its feasibility that can also be useful to other countries.

Conclusions

The PSC is a feasible and relevant tool to support egalitarian follow-up and identify patients who can benefit from targeted interventions after stroke. HPs' stroke expertise , room for dialogue, and caring for identified needs were raised as important issues to consider when using the PSC. Nutrition, sexuality, driving, work and fatigue were areas mentioned that might need to be adressed within the discussions by HPs using the checklist. The PSC can facilitate patients in expressing their needs, enhancing their ability to participate in decision-making.

Acknowledgements

The authors thank the health professionals and patients, for sharing their experiences. The authors also thank Dr Kate Bramley-Moore translation help regarding quotations from the transcribed interviews.

Author contribution

EK, ÅL-N, and KSS contributed to the design of the study. EK conducted the interviews and analyzed the data together with GC, involving KSS and ÅL-N in the final stages of the analysis. EK wrote the first version of the manuscript, which was reviewed by GC, ÅL-N KSS. All four authors contributed to and approved the final manuscript.

Funding

The study was funded in part by the Swedish Stroke Association; the Local Research and Development Board for Gothenburg and Södra Bohuslän; the Swedish Heart and Lung foundation; the Swedish Brain Foundation; an unconditional grant from Allergan; the Hjalmar Svenssons Foundation; Neurological Foundation; Rune and Ulla Amlövs Foundation; Per-Olof Ahls Foundation; and John and Brit Wennerströms Foundation. The study was supported

For peer review only - http://bmjopen.bmj.com/site/about/guidelines.xhtml

by grants from the Swedish state under the agreement between the Swedish government and the county councils, the ALF agreement (ALFGBG-719 80).

Competing interests

None declared.

Data sharing

Due to ethical restrictions, data are available upon request. Researchers can submit requests for data to the authors (contact: ks.sunnerhagen@neuro.gu.se). Complete data from interviews cannot be made publicly available for ethical and legal reasons, according to the Swedish regulations http://www.epn.se/en/start/regulations/. Public availability would compromise participant privacy or confidentiality. Upon request a list of condensed meaning units or codes can be made available after removal of information that may risk the confidentiality of the participants. To access data please contact the first author: (emma.kjork@neuro.gu.se).

For peer review only

REFERENCES

1. Crichton SL, Bray BD, McKevitt C, et al. Patient outcomes up to 15 years after stroke: survival, disability, quality of life, cognition and mental health. *J Neurol Neurosurg Psychiatry* 2016;87(10):1091-8. doi: 10.1136/jnnp-2016-313361

2. Ullberg T, Zia E, Petersson J, et al. Changes in functional outcome over the first year after stroke: an observational study from the Swedish stroke register. *Stroke* 2015;46(2):389-94. doi: 10.1161/STROKEAHA.114.006538

3. Ullberg T, Zia E, Petersson J, et al. Perceived Unmet Rehabilitation Needs 1 Year After Stroke: An Observational Study From the Swedish Stroke Register. *Stroke* 2016;47(2):539-41. doi: 10.1161/STROKEAHA.115.011670

4. Carlsson GE, Moller A, Blomstrand C. Consequences of mild stroke in persons <75 years -- a 1-year follow-up. *Cerebrovasc Dis* 2003;16(4):383-8. doi: 10.1159/000072561

5. Cumming TB, Marshall RS, Lazar RM. Stroke, cognitive deficits, and rehabilitation: still an incomplete picture. *Int J Stroke* 2013;8(1):38-45. doi: 10.1111/j.1747-4949.2012.00972.x

6. Levasseur M, Carrier A. Integrating health literacy into occupational therapy: Findings from a scoping review. *Scand J Occup Ther* 2012;19(4):305-14. doi: 10.3109/11038128.2011.588724

7. Mackey LM, Doody C, Werner EL, et al. Self-Management Skills in Chronic Disease Management: What Role Does Health Literacy Have? *Med Decis Making* 2016;36(6):741-59. doi: 10.1177/0272989X16638330 [published Online First: 2016/04/08]

8. Kirkevold M. The unfolding illness trajectory of stroke. *Disabil Rehabil* 2002;24(17):887-98. doi: 10.1080/09638280210142239

9. Tistad M, Ytterberg C, Tham K, et al. Poor concurrence between disabilities as described by patients and established assessment tools three months after stroke: a mixed methods approach. *J Neurol Sci* 2012;313(1-2):160-6. doi: 10.1016/j.jns.2011.08.038 [published Online First: 2011/09/23]

10. Ekman I, Swedberg K, Taft C, et al. Person-centered care—Ready for prime time. *Eur J Cardiovasc Nurs* 2011;10(4):248-51.

11. Philp I, Brainin M, Walker MF, et al. Development of a poststroke checklist to standardize follow-up care for stroke survivors. *J Stroke Cerebrovasc Dis* 2013;22(7):e173-80. doi: 10.1016/j.jstrokecerebrovasdis.2012.10.016

For peer review only - http://bmjopen.bmj.com/site/about/guidelines.xhtml

12. Ward AB, Chen C, Norrving B, et al. Evaluation of the Post Stroke Checklist: a pilot study in the United Kingdom and Singapore. *Int J Stroke* 2014;9 Suppl A100(A100):76-84. doi: 10.1111/ijs.12291
13. Iosa M, Lupo A, Morone G, et al. Post Soft Care: Italian implementation of a post-stroke checklist software for primary care and identification of unmet needs in community-dwelling patients. *Neurol Sci* 2017;39(1):135-39. doi: 10.1007/s10072-017-3140-1
14. Hotter B, Padberg I, Liebenau A, et al. Identifying unmet needs in long-term stroke care using in-depth assessment and the Post-Stroke Checklist – The Managing Aftercare for Stroke (MAS-I) study. *European Stroke Journal* 2018;3(3):237-45. doi: 10.1177/2396987318771174
15. Krueger RA. Focus groups: A practical guide for applied research. 5th ed. London: Sage publications 2014.
16. Ivanoff SD, Hultberg J. Understanding the multiple realities of everyday life: basic assumptions in focus-group methodology. *Scand J Occup Ther* 2006;13(2):125-32.
17. Craig P, Dieppe P, Macintyre S, et al. Developing and evaluating complex interventions: the new Medical Research Council guidance. *BMJ* 2008;337:a1655. doi: 10.1136/bmj.a1655
18. Beaton DE, Bombardier C, Guillemin F, et al. Guidelines for the process of cross-cultural adaptation of self-report measures. *Spine (Phila Pa 1976)* 2000;25(24):3186-91. [published Online First: 2000/12/22]
19. Tong A, Sainsbury P, Craig J. Consolidated criteria for reporting qualitative research (COREQ): a 32-item checklist for interviews and focus groups. *Int J Qual Health Care* 2007;19(6)
20. Malterud K, Siersma VD, Guassora AD. Sample Size in Qualitative Interview Studies: Guided by Information Power. *Qual Health Res* 2015 doi: 10.1177/1049732315617444 [published Online First: 2015/11/29]
21. Paul SL, Srikanth VK, Thrift AG. The large and growing burden of stroke. *Curr Drug Targets* 2007;8(7):786-93. [published Online First: 2007/07/17]
22. Stevens E, Emmett E, Wang DY, et al. The Burden Of Stroke In Europe Report London: SAFE Stroke Alliance For Europe 2017.
23. Norrving B, Barrick J, Davalos A, et al. Action Plan for Stroke in Europe 2018–2030. *European Stroke Journal*;0(0):2396987318808719. doi: 10.1177/2396987318808719

For peer review only - http://bmjopen.bmj.com/site/about/guidelines.xhtml

24. Shannon RL, Forster A, Hawkins RJ. A qualitative exploration of self-reported unmet need one year after stroke. *Disabil Rehabil* 2016;38(20):2000-7. doi: 10.3109/09638288.2015.1107784

25. Williams MV, Davis T, Parker RM, et al. The role of health literacy in patient-physician communication. *Fam Med* 2002;34(5):383-9.

26. Silverman J, Kurtz S, Draper J. Skills for communicating with patients. 3rd ed. London: CRC Press 2016.

27. Reinholdsson M, Palstam A, Sunnerhagen KS. Prestroke physical activity could influence acute stroke severity (part of PAPSIGOT). *Neurology* 2018;91(16):e1461-e67. doi: 10.1212/WNL.0000000000006354 [published Online First: 2018/09/21]

For peer review only

For peer review only - http://bmjopen.bmj.com/site/about/guidelines.xhtml

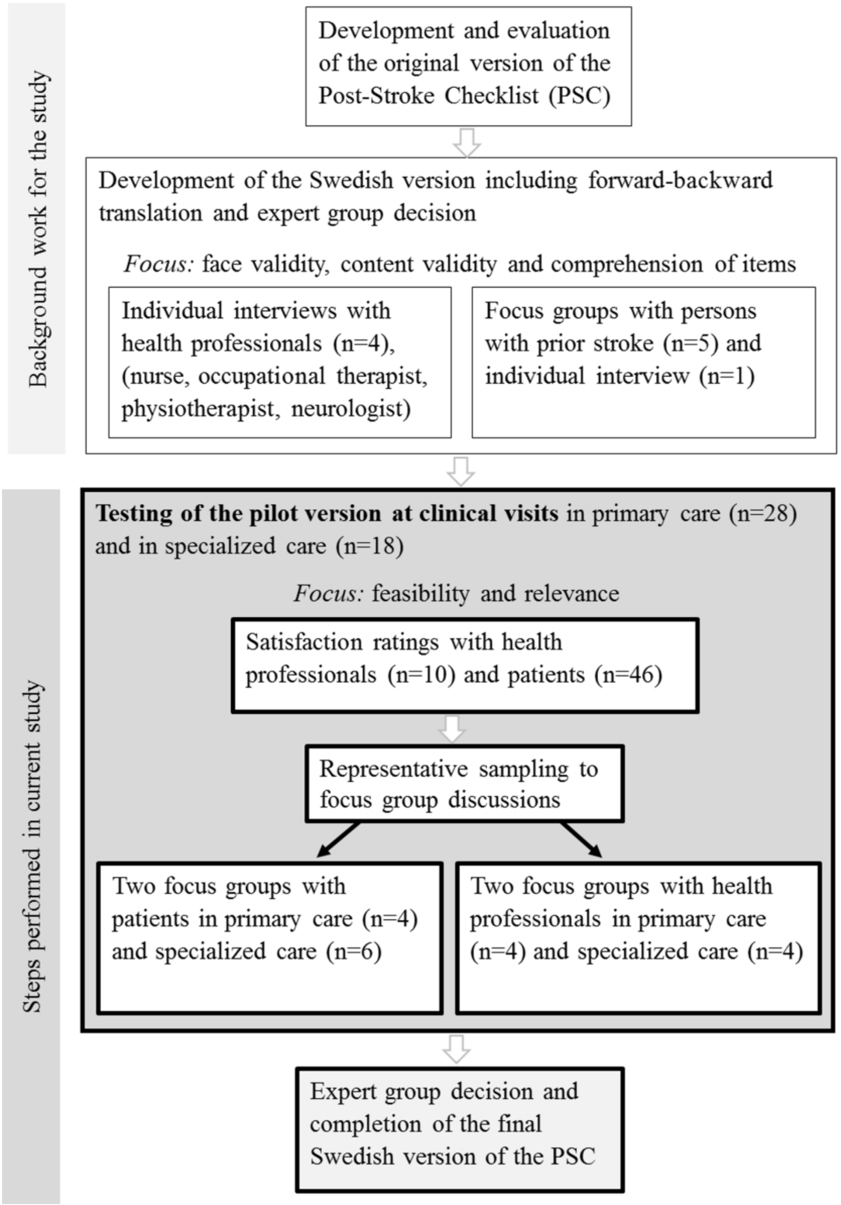

Figure 1. The steps included in the validation and cross-cultural adaptation of the Swedish version of the PSC including background work and current study.

199x267mm (300 x 300 DPI)

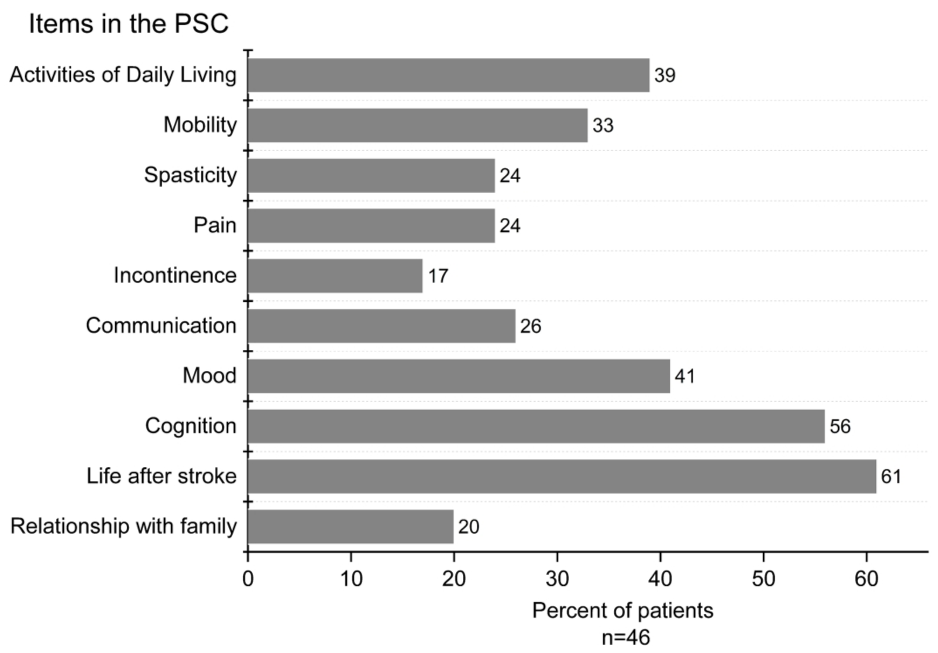

Figure 2. Percentages of patients with identified problems in each Post-Stroke Checklist item (n=46).

229x160mm (300 x 300 DPI)

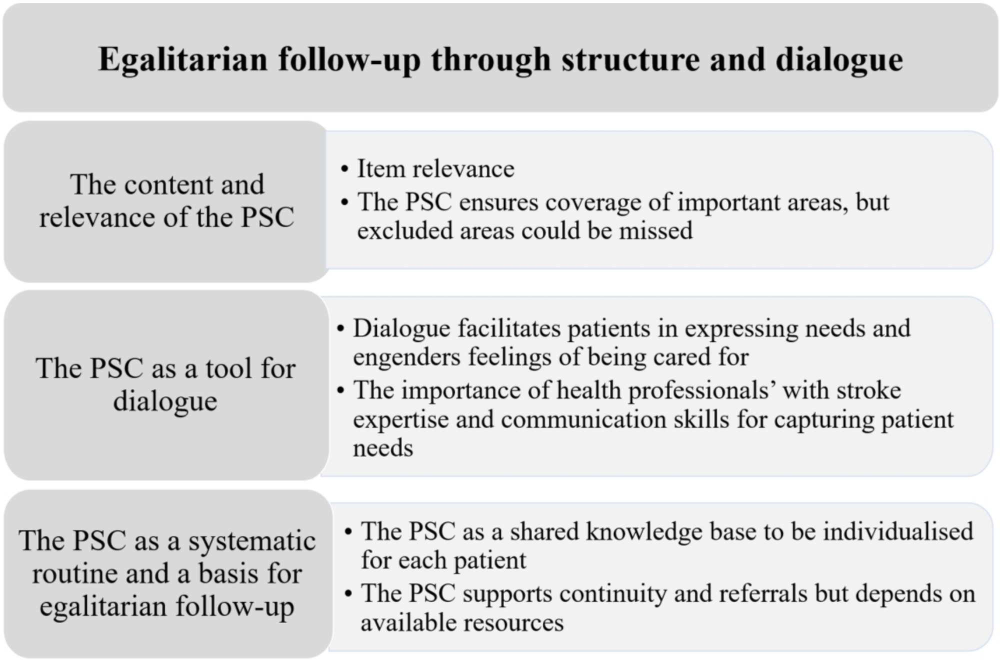

Figure 3. Themes and subthemes derived from the focusgroup discussions with patients and health professionals regarding experiences of using the Post-Stroke Checklist.

169x111mm (300 x 300 DPI)

Appendix 2 - COREQ 32-item checklist

**No. Item**	**Guide questions/description**	**Comments**
**Domain 1: Research team and reflexivity**		
Personal characteristics		
1. Interviewer/facilitator	Which author/s conducted the interview?	EK (p.6)
2. Credentials	What were the researcher's credentials? *e.g., PhD, MD*	3 Occupational Therapists (1 associate professor, 1 PhD, and 1 MS, PhD student) 1 Medical doctor, (Professor)
3. Occupation	What was their occupation at the time of the study?	Researchers
4. Gender	Was the researcher male or female?	4 female (p. 6-7)
5. Experience and training	What experience or training did the researchers have?	> 20 years in stroke rehab, Qualitative research (p. 7)
Relationship with participants		

6. Relationship established	Was a relationship established prior to study commencement?	The moderator had met some of the health professionals in work related situations.
7. Participant knowledge of the interviewer	What did the participants know about the researcher? *e.g., personal goals, reasons for doing the research*	Broad outlines given
8. Interviewer characteristics	What characteristics were reported about the interviewer/facilitator? *E.g., bias, assumptions, reasons and interests in the research topic*	(p. 5)
**Domain 2: study design**		
Theoretical framework		
9. Methodological orientation and Theory	What methodological orientation was stated to underpin the study? *e.g. grounded theory, discourse analysis, ethnography, phenomenology, content analysis*	Focus group according to Kreuger, underpinned by a methodology based on Social constructivism (p. 4)
Participant selection		

For peer review only

10. Sampling	How were participants selected? *e.g. purposive, convenience, consecutive, snowball*	Purposive (p. 5)
11. Method of approach	How were participants approached? e.g. *face-to-face, telephone, mail, email*	Face to face and by telephone (p. 5)
12. Sample size	How many participants were in the study?	In total, 18 (patients/HPs) participated in the focus groups (see table 2, p 7)
13. Non-participation	How many people refused to participate or dropped out? Reasons?	One dropped out because of a scheduled medical examination (p. 9)
Setting		
14. Setting of data collection	Where was the data collected? *e.g. home, clinic, workplace*	Clinical setting (p. 5)
15. Presence of non-participants	Was anyone else present besides the participants and researchers?	No (p. 6)
16. Description of sample	What are the important characteristics of the sample? *e.g. demographic data, date*	People with stroke in a late phase of stroke recovery (p. 7, table 2)
Data collection		

For peer review only

17. Interview guide	Were questions, prompts, guides provided by the authors? Was it pilot tested?	A interview guide was provided (p.6). A pilot testing was conducted (p. 5).
18. Repeat interviews	Were repeat interviews carried out?	No, each group met once (p. 6)
19. Audio/visual recording	Did the researchers use audio or visual recording to collect the data?	Audio (p. 6)
20. Field notes	Were field notes made during and/or after the interview or focus group?	Yes
21. Duration	What was the duration of the interviews or focus group?	Approximately 1.5 hours (p. 6)
22 Data saturation	Was data saturation discussed?	Yes (p. 6)
23. Transcripts returned	Were transcripts returned to participants for comment and/or correction?	No
**Domain 3: analysis and findings**		
Data analysis		
24. Number of data coders	How many data coders coded the data?	Two (EK and GC) (p. 6)
25. Description of the coding tree	Did authors provide a description of the coding tree?	Yes.
26. Derivation of themes	Were themes identified in advance or derived from the data?	Derived from the data (p. 6)

27. Software	What software, if applicable, was used to manage the data?	Nvivo (p. 6)
28. Participant checking	Did participants provide feedback on the findings?	No, not specifically, but a general presentation (p. 5)
Reporting		
29. Quotations presented	Were participant quotations presented to illustrate the themes/findings? Was each quotation identified? *e.g. participant number*	Yes (Results)
30. Data and findings consistent	Was there consistency between the data presented and the findings?	Yes (Results and Discussion)
31. Clarity of major themes	Were major themes clearly presented in the findings?	Yes (Figure 3 and results)
32. Clarity of minor themes	Is there a description of diverse cases or discussion of minor themes?	Yes (Results and Discussion)

For peer review only

BMJ Open

Experiences using the Post-Stroke Checklist in Sweden with a focus on feasibility and relevance: a mixed-method design

Journal:	*BMJ Open*
Manuscript ID	bmjopen-2018-028218.R2
Article Type:	Research
Date Submitted by the Author:	21-Feb-2019
Complete List of Authors:	Kjörk, Emma K; University of Gothenburg, Institute of Neuroscience and Physiology, Department of Clinical Neuroscience<br>Carlsson, Gunnel; University of Gothenburg, Institute of Neuroscience and Physiology, Department of Clinical Neuroscience<br>Sunnerhagen, Katharina; University of Gothenburg, Institute of Neuroscience and Physiology, Department of Clinical Neuroscience<br>Lundgren-Nilsson, Asa; Gothenburg University, Institute of Neuroscience and Physiology, Department och Clinical Neuroscience
**Primary Subject Heading**:	Cardiovascular medicine
Secondary Subject Heading:	Evidence based practice, Health services research, Rehabilitation medicine
Keywords:	long-term care, Feasibility, Focus groups, Follow-up, Standard of Care, Stroke < NEUROLOGY

SCHOLARONE™
Manuscripts

Experiences using the Post-Stroke Checklist in Sweden with a focus on feasibility and relevance: a mixed-method design

Emma K Kjörk* OT, MS[1, 2], Gunnel Carlsson OT, PhD[1], Katharina S Sunnerhagen MD, PhD, Prof[1] and Åsa Lundgren-Nilsson OT, PhD[1]

[1]Institute of Neuroscience and Physiology, Dep of Clinical Neuroscience, Sahlgrenska Academy at University of Gothenburg, Gothenburg, Sweden
[2]Department of Occupational Therapy and Physiotherapy, Sahlgrenska University Hospital, Gothenburg, Sweden

* Corresponding author:
Emma K Kjörk
Email address: emma.kjork@neuro.gu.se
Telephone: +46313422803
Postal address:
Institute of Neuroscience and Physiology
Dep of Clinical Neuroscience, Rehabilitation medicine
Per Dubbsgatan 14, fl. 3, 413 45 Gothenburg, Sweden

Word count Abstract: 300 words
Word count Main manuscript: 3993 words
Number of tables: 3
Number of figures: 3

For peer review only

For peer review only - http://bmjopen.bmj.com/site/about/guidelines.xhtml

Experiences using the Post-Stroke Checklist in Sweden with a focus on feasibility and relevance: a mixed-method design

ABSTRACT

Objective The wide range of outcomes after stroke emphasises the need for comprehensive long-term follow-up. The aim was to evaluate how people with stroke and health professionals (HPs') perceive the use of the Post-Stroke Checklist (PSC), with a focus on feasibility and relevance.

Design An exploratory design with a mix of qualitative and quantitative methods.

Setting Outpatient care at a university hospital and primary care centres in western Sweden.

Participants Forty-six consecutive patients (median age, 70; range, 41–85; 13 women) and ten health professionals (median age 46; range, 35-63; 7 women).

Results Most patients (87%) had one or more problems identified by the PSC. The most common problem areas were life after stroke (61%), cognition (56%), mood (41%), and activities of daily living (39%). Three organisational themes emerged from the focus group discussions. The perception of *the content and relevance of* the PSC was that common post-stroke problems were covered but that unmet needs still could be missed. Identifying needs was facilitated when using the *PSC as a tool for dialogue.* The dialogue between the patient and HP as well as HPs stroke expertise was perceived as important. The PSC was seen as *a systematic routine and a base for egalitarian follow-up*, but participants stressed consideration given to each individual. Addressing identified needs and meeting patient expectations were described as challenging given available healthcare services.

Conclusions The PSC is a feasible and relevant tool to support egalitarian follow-up and identify patients who could benefit from targeted post-stroke interventions. Stroke expertise, room for dialogue, and caring for identified needs emerged as important issues to consider when using the PSC. Nutrition, sexuality and fatigue were areas mentioned that might need to be addressed within the discussions. The PSC can facilitate patients in expressing their needs, enhancing their ability to participate in decision-making.

For peer review only - http://bmjopen.bmj.com/site/about/guidelines.xhtml

Keywords Stroke, Rehabilitation, Standard of care, Feasibility, Focus groups, Follow-up

Strengths and limitations (bullet points)

- A strength of this study is having patients as partners throughout the project, from the translation process to participation in the focus groups.

- The mix of methods made it possible to explore the feasibility of the PSC from different perspectives and at different levels compared to previous research.

- A limitation is the lack of data collected from people with severe stroke, although this lack is typical of this naturalistic design and representative for the Swedish stroke population.

- A study strength is the heterogeneous population from different outpatient settings, including a range of ages, stroke characteristics, and education levels, and health professionals with a range of experience in stroke care.

- The study was conducted in a Swedish context, and the transferability of the findings in other cultural contexts is not known.

For peer review only

INTRODUCTION

Post-stroke impairments often have long-term negative consequences for social relationships, dependence in daily life, and quality of life.[1] Perceived unfulfilled rehabilitation needs and changes in functioning during the first year after stroke[2 3] indicate the need for systematic long-term follow-up. Furthermore, a subtle decline in cognition as well as emotional problems[4 5] can easily be overlooked, leading to difficulty in accessing healthcare services.[6 7]

The adaptation process after stroke is long. In later phases, the focus changes from the stroke itself to more familiar aspects of daily life,[8] which may not be sufficiently targeted in current practice. Concordance is poor between perceived problems and problems detected by standardised assessments. Accordingly, dialogue should complement assessments to ensure that health services are based on patient needs,[9] in line with a person-centred approach.[10]

The Post-Stroke Checklist (PSC)[11] was developed to identify long-term care needs and facilitate referrals.[11 12] Although the PSC has been found to be feasible and useful,[12-14] knowledge is lacking about the perspectives of patients and health professionals (HPs) regarding its use in the Swedish health care context. The aim of this study was to evaluate how people with stroke and HPs perceive the use of the PSC, with a focus on feasibility and relevance.

METHODS

Study design

The study has an exploratory design. To capture the feasibility of using the PSC, we combined it with a satisfaction questionnaire and focus group discussions,[15 16] in line with guidelines for complex interventions.[17] This study is part of a validation and cultural adaptation process[18] of the PSC in Sweden (figure 1). By combining data collection methods, we expected to gain a deeper understanding of using the PSC as a tool to structure follow-up. The underpinning methodology in the focus groups is based on social constructivism.[16] Consolidated criteria for reporting qualitative research (i.e., COREQ guidelines)[19] were followed for reporting qualitative data.

[Insert figure 1 near here]

For peer review only

Patient and public involvement

This study explores how people with stroke experience the PSC. People from the Swedish Stroke Association (patient association) were involved in the translation process, the pilot testing of the interview guide, and focus group discussions and have been given a presentation of preliminary results.

Participants

Participants were consecutively recruited while at a clinical visit in primary care or stroke specialised outpatient care at a university hospital, February 2015 to October 2015. The inclusion criterion was having had a stroke, regardless of the time of onset. The number of patients included was in accordance with a previous study[12] and principles for cross-cultural adaptation suggesting approximately 40.[18] Patients were excluded if cognitive impairment or insufficient knowledge of Swedish would have made the response to the PSC items unreliable. HPs from different clinics were invited to participate and selected to represent different professions. A purposive sampling was used with the attempt to achieve heterogeneity and homogeneity in the focus groups.[15 16] Written informed consent was obtained from all participants, and the regional ethical review board in Gothenburg approved the study (no. 521-14).

Data collection

The PSC[11] has 11 items and is intended to identify post-stroke problems. It was developed by a multi-professional group of stroke experts, according to a Delphi process. Problem areas were chosen for having the greatest impact on patient quality of life that could be addressed with evidence-based interventions. The PSC includes secondary prevention, activities of daily living (ADLs), mobility, spasticity, pain, incontinence, communication, mood, cognition, life after stroke, and relationship with family. A response scale includes ‘yes’ and ‘no’ and recommended referrals adjacent to each problem area.[11]

Data collection was conducted in outpatient clinical facilities in two steps. For step 1, the HP administered the PSC to patients at a regular clinical visit (proxy responses were allowed). No additional training was given beyond the general instructions on the PSC. Participants were asked to reflect on the usefulness of the PSC as the basis for focus group discussions. Patients and HPs assessed satisfaction with the PSC after each visit through a satisfaction questionnaire with questions analogous to those used in a previous study.[12] The answers were rated on a Likert scale of 1–5, where 1 indicated not satisfied and 5 completely

For peer review only - http://bmjopen.bmj.com/site/about/guidelines.xhtml

satisfied. To ensure anonymity, patient responses were collected in an envelope. Demographic data (time since index stroke, age, sex), time to administration of the PSC, and HP profession were registered. In addition, if any referrals were made it was registered as yes/no for each patient without specification of what kind of referrals or standardised "actions" to be taken. Of the patients who gave informed consent, additional patient characteristics (such as type of stroke, aphasia, ADL dependency) were collected retrospectively from their medical records.

For step 2, the staff invited all participants during the follow-up to join in a focus group discussion. A set time of approximately 1-2 months was given between the visit and the focus group discussions. The first author (EK) telephoned participants willing to participate and sent them the study information letter, time for appointment, and a copy of the PSC. In total, four focus groups were conducted using a question guide.[15] Each focus group met once for approximately 1.5 hours. The meeting was recorded and transcribed verbatim. Initially in the focus groups, the importance of bringing up different opinions was emphasised. At the end, an oral overview was presented to ensure that participant contributions were as they intended.

Data analysis

Data gathered from PSC items, questionnaires, and demographic information were analysed using descriptive statistics with Statistical Package for the Social Sciences version 24. Data gathered from focus groups were analysed following Kreuger.[15] The aim of the analysis process was to describe the participants' perceptions and experiences based on the aim of the study.

Immediately after each focus group discussion, a written summary was created. The transcripts were coded using the computer software NVivo. First, the transcripts were read in their entirety to allow familiarity with the content as a whole, and discussions relevant to the aim were identified. Second, transcripts were systematised into categories based on similarities and differences in the discussions. Third, a descriptive summary was made for each category, adhering as closely as possible to the content of the raw data. Finally, these summaries in combination with selected quotes served as the basis for the interpretation and presented a deeper insight into the findings. In the analysis process, identified patterns were compared and contrasted across all four groups, resulting in an overarching thematic structure (for examples of the coding tree and themes see table 1). Quotations that showed the ongoing discussions[16] were selected to illuminate the results. Based on sampling strategies[20] and when similar discussions recurred in all groups[15], data gathering stopped.

For peer review only - http://bmjopen.bmj.com/site/about/guidelines.xhtml

The first author (PhD student, OT, woman) was the moderator and performed most of the analysis. Multiple coding, continuous interpretation of data and discussion of the emerging themes were completed together with the second author (PhD, OT, woman) to ensure accuracy of the analysis. The first author had knowledge about the study topic and the second author in qualitative methods. Both have conducted interviews previously. The third author (PhD, MD, woman) and last author (PhD, OT, woman) contributed with knowledge concerning revising and refining the themes. All authors have at least 20 years of experience in stroke rehabilitation.

Table 1. Examples illustrating the coding tree.

Quote	Code	Subtheme
P4:"It has a lot to do with the competence of the person who's asking the questions so they can do the thinking to squeeze it all in" (Group 1 Patients)	The professionals' expertise and reasoning	The importance of HPs' with stroke expertise and communication skills for capturing patient needs
P2: "So, I think it has just been positive, and it is also done so quickly".<br>P1: "That's also a positive".<br>P2: "Yeah, it's fast, but you can also develop it as much as you want. But asking the questions doesn't take too long".<br>Moderator: "Is it quick?"<br>P1: "Yes. It also depends on what answers you get".<br>P2: "Yes".<br>(Group 4. Health professionals)	The administration of the PSC can be adapted, quickly done or more in-depth	The PSC supports continuity and referrals but depends on available resources

RESULTS

Study group

The PSC was used in connection with a clinical visit in 46 patients. All patients lived in their homes. Most of them (65%) had experienced a stroke within 3 months at enrolment. The median time for hospitalisation was 8 days. Stroke severity at stroke onset was mild, with a

For peer review only - http://bmjopen.bmj.com/site/about/guidelines.xhtml

median of 2 according to National Institutes of Health Stroke Scale. Table 2 shows the characteristics of the participants and the focus groups.

Table 2. Characteristics of patients and health professionals in the clinical outpatient visits and the focus group discussions. Data are presented as number of persons (n) or median and range.

**Patients**	Clinical visit (n=46)	Focus group 1 (n=4)	Focus group 2 (n=6)
Primary care, rural		x	
Specialized care, urban			x
Age at inclusion	70 (41-85)	71 (58-78)	74 (45-76)
Sex, male	33, 72%	4	5
Education			
Mandatory	20	1	4
High School	13	1	1
University	8	2	1
Months since stroke	3 (1-84)	20 (3-84)	3 (1-6)
Working at stroke onset (yes)	13	2	1
Length of hospitalization, (days)	8 (2-120)	11 (5-82)	8 (4-11)
History of stroke (yes)	9	1	3
Stroke characteristics			
Ischemic/Haemorrhagic	36/5	4/0	4/2
Right/ left/ posterior/ bilateral	19/16/5/2	3/1/0/0	3/2/1/0
NIHSS	2 (0-16)	4 (3-10)	2 (1-6)
Aphasia (yes)	9	0	1
Neglect (yes)	4	1	0
At discharge			
ADL independency (yes)	34	3	6
Wheel-chair use (yes)	4	1	0
**Health Professionals**	Clinical visit (n=10)	Focus group 3 (n=4)	Focus group 4 (n=4)
Age	46 (35-63)	43 (37-46)	46 (35-55)
Primary care, rural			x
Specialized care, urban		x	
Sex, male	3, 30%	0	1
Nurse/ OT/ Physician	4/1/5	3/0/1	0/1/3
Stroke experience (years)			
≤5/ 5-10/ 10	2/2/6	0/1/3	2/1/1

Abbreviations: NIHSS=National Institutes of Health Stroke Scale, ADL= Activities of Daily Living, OT= Occupational Therapist.

(Missing data from medical records (n=4).

For peer review only - http://bmjopen.bmj.com/site/about/guidelines.xhtml

Feasibility of the PSC

Forty patients (87%) had one or more problems identified by the PSC (figure 2). 'Life after stroke' was most common (61%), followed by cognition (56%) and mood changes (41%). A median of four problem areas per patient (range, 0–9; interquartile range [IQR], 1–5) was identified; the median in specialised care was 3 (IQR, 1–5), and in primary care, it was 4 (IQR, 1–5). Only six (13%) patients reported no problems. Most patients (70%) acknowledged having received information about secondary prevention. Referrals were registered in eight cases, slightly more often in primary care (n=6) than in specialised care (n=2). The time taken to administer the PSC was ≤15 minutes for 52%, ≤30 minutes for 43%, and ≥45 minutes for 5%.

[Insert figure 2 near here]

Four focus groups were conducted, and their characteristics are shown in table 2. One woman dropped out because of a scheduled medical examination. The focus group discussions revealed that the PSC structure in combination with room for dialogue could support egalitarian follow-up and identification of needs. A main theme and three organisational themes emerged in these discussions (figure 3).

[Insert figure 3 near here]

The content and relevance of the PSC

Item relevance

The items included in the PSC were considered relevant to all groups. Because stroke affects persons differently, participants found it valuable that the PSC covers a broad spectrum of problems, although not all problems are relevant to every person with stroke.

The PSC ensures coverage of important areas, but excluded areas could be missed

Both patients and HPs stated that some issues might be overlooked if not specifically stated in the PSC; such as nutrition, sexuality, vision, irritability and driving. The HPs discussed the appropriate amount of problem areas in the PSC. They wanted more areas yet preferred the checklist to be short and complemented by profession-specific assessments when needed. Some participants appreciated the recurring phrase "since your stroke", but others preferred to hear/say it once at the beginning of the visit. Patients perceived the PSC as easy to

For peer review only - http://bmjopen.bmj.com/site/about/guidelines.xhtml

understand, while HPs expressed a concern about misunderstandings, especially for the items ‘secondary prevention’ and ‘spasticity’.

The PSC as a tool for dialogue

Patients and HPs both emphasised the need for dialogue to create mutual understanding. HPs described that knowledge and experience affected their ability to detect problems, while patients described differences in their ability to communicate problems.

Dialogue facilitates patients in expressing needs and engenders feelings of being cared for

Patients said that the PSC questions facilitated dialogue, leading to a greater likelihood that important areas would be elucidated and discussed. Memory problems, lack of initiative, fatigue, or being less talkative were mentioned as barriers to dialogue that the PSC could address. The PSC gave clear direction for the structure of the dialogue and accordingly facilitating identification of problems. Nevertheless, using the PSC in combination with dialogue was seen as important. Patients stated that they might need time for consideration before answering the PSC questions, time that was often not given within the limits of the visits.

Generally, participants thought patients should have the opportunity to talk to a professional about stroke-related concerns and stated that the PSC could facilitate this exchange. Patients expressed that the PSC covers areas centring on them as a person, which made them feel cared for. The understanding of the HPs was that relatives often complemented information concerning problems that patients might neglect or forget to mention:

> *P4: “I think there is a great deal of importance to how much you are affected by the stroke. The more you are affected, the harder it is to think about the different facets of it”* (the areas in the PSC). (Strongly agreed upon)
> (Group 1. Patients)

The importance of HPs’ with stroke expertise and communication skills for capturing patient needs

To ensure that problems would be fully addressed, a professional’s competence in stroke was seen as key by the participants. HPs feared that lack of HP’s with stroke expertise might lead

For peer review only - http://bmjopen.bmj.com/site/about/guidelines.xhtml

to problems going unrecognised. The PSC was seen as an asset as well as a barrier to dialogue. If too much focus was placed on posing the questions, participants experienced a decreased interaction. Sensitivity from the HPs and use of additional questions was seen as essential (e.g. work issues, within the item 'life after stroke'):

> *P7: "No, there's only benefits, but it depends on how you use it, and if the staff think it is meaningful, so it's not just checked off. Rather that you have the opportunity to cover the things that each point is actually about".*
> *P8: "Yeah, all the stuff that's crazy [difficulties after the stroke], follow up that stuff."*
> *Moderator: "That you have time to follow up what is included in the point? Can you elaborate on that?"*
> *P7: "Yes, it's the topic this question is about, that you can elaborate on if you want to (...) the person with the checklist shouldn't be bound to it 100% and slavishly follow it, but understand signals from the patient and talk more broadly and connect it to the other things that depend upon it". (agreement)*
> *P9: "Absolutely".*
> *P7: "Otherwise, it just becomes mechanical; you can't be just like a computer asking questions".*
> (Group 2. Patients)

There were conflicting opinions about how to apply the questions in the PSC. Experienced HPs preferred to use it as a supplement for memory within a free dialogue. In contrast, inexperienced HPs perceived the specific questions as good to assume and a basis for leading into other related concerns (e.g. fatigue):

> *P5: "It's more about if you think that you should use standardised things for everyone, even for primary healthcare/outpatient care, I don't think that really works".*
> *P6: "Exactly, that's the question, should you use the checklist just as it is, or should you use it for your own part and remember. That's the thing, because you can then approach each patient differently and get it all. But asking the exact same questions for each patient, I agree, that's really hard to do".*
> (Group 3. HPs)

For peer review only

For peer review only - http://bmjopen.bmj.com/site/about/guidelines.xhtml

PSC as a systematic routine and a basis for egalitarian follow-up

The PSC as a shared knowledge base to be individualised for each patient

The PSC was considered to increase knowledge about stroke and secure an egalitarian follow-up, especially for inexperienced HPs and patients with limited ability to express their needs. Even when the PSC was used, lack of HPs with stroke expertise and limited knowledge about opportunities for referrals were perceived as an obstacle to egalitarian follow-up. One suggestion was to add local referral opportunities and access to scientific references in conjunction with the PSC. Factors such as comorbidities and time since stroke must be taken into account because they could affect responses to PSC items.

The PSC supports continuity and referrals but depends on available resources

Participants addressed the need for regular follow-ups and considered the PSC to be a useful tool and basis for referrals. The use of the PSC was seen as a rapid way to cover the problem areas if only the questions were used, but when supplementary questions were needed, the time need also increased. One concern, especially among the physicians, was the time taken to administer the PSC in addition to their ordinary routines. HPs emphasised that the use of the PSC should be beneficial for the patients and in accordance with time limits and referral opportunities. Some considered it a bit rigid to go through all items if the patient experienced no problems, although it was observed that it could be completed quickly.

To enable preparation beforehand and make visits more time-efficient, a patient version of the PSC was proposed. Participants strongly emphasised that problems identified by the PSC should lead to appropriate intervention and not only an evaluation of current status:

> *P11: "The risk is you might get a false sense of security though. So, someone has asked the question, and I have answered "yes" to this question; so I then expect something to happen".*
>
> (agreement from the others)
>
> *P11: "It's like, that's what decides the quality of what happens with the measures (...). It should end with me knowing how this information is taken and handled, what happens now. Not just that you do it and then that's great. (mumbles) Is it like statistics or what?"*
>
> (Group 1. Patients)

For peer review only - http://bmjopen.bmj.com/site/about/guidelines.xhtml

Lack of opportunities for interventions as well as knowledge gaps were expressed by HPs as difficulties in meeting these expectations. A specific dimension of this problem was mentioned as leading to a risk of avoidance of discussing certain items.

Table 3. Evaluation of the use of Post-Stroke Checklist (PSC) based on satisfaction ratings (Likert 1-5) by patients and health professionals.

Satisfaction with:	Patients (n=46) median (IQR)	Health professionals (n=10) median (IQR)
Overall assessment where PSC was used	5 (4-5)	4 (3-4)
Identification of needs	5 (4-5)	-
Identification of need (for each patient)	-	3 (3-4)
Confidence in receiving support	5 (4-5)	-
Guidance for referrals and treatment	-	3 (2-4)

Abbreviations: IQR= interquartile range

For peer review only

By combining the results derived by different methods, additional aspects of the analysis can be demonstrated. The patients evaluated the satisfaction with the PSC as high (table 3). In addition, the focus group analyses gave insights into a wide range of factors that affect satisfaction, and its feasibility was exemplified by the importance of dialogue (figure 3). HP satisfaction with the PSC varied among patients (table 3). Participants perceived it as important to adapt the use of the PSC to individual aspects. Some differences regarding individual prerequisites are displayed in table 2, and others are mentioned in the qualitative analyses. HP stroke experience varied, especially in the primary care settings (table 2). In the focus group discussions, HPs with stroke expertise was perceived as important if subtle problems were to be properly recognised.

DISCUSSION

The PSC is a relevant and feasible tool to identify patients who can benefit from targeted interventions, as noted by people with stroke and HPs. The original purpose of the PSC was to be an easy-to-use tool to detect post-stroke problems, as well as a support for guiding referrals.[11] This study brings out an awareness about how follow-up through the PSC could be enhanced by user perceptions and suggested strategies. This knowledge could add important insights when implementing the PSC in line with the World Stroke Organization recommendations. The focus group discussions raised issues concerning prerequisites when

For peer review only - http://bmjopen.bmj.com/site/about/guidelines.xhtml

using the PSC. These include HP with stroke expertise, room for dialogue, and how the identified needs were addressed.

The wide range of post-stroke problems identified in the present study demonstrated the relevance of the PSC, with a median of four problems per patient. Of note, reported problems on specific PSC items differ considerably; ‘life after stroke’, cognition, and continence vary when comparing among countries.[12-14] Comparison should be made with caution since the groups studied differ with respect to e.g. case-mix, sampling strategies and inclusion criteria in the studies. Further, based on issues raised in the focus group discussions, likely causes of these discrepancies in reported problems could be HP stroke expertise, opportunity for a dialogue, and time limits on the administration of the PSC. Comorbidities also could affect responses to the PSC items due to respondents not being able to consider whether the problems are stroke related or not. Nevertheless, the wide range of identified problems alongside participant perceptions in this study stresses the relevance of using the PSC in clinical practice. The long-term consequences after stroke emphasise the need for a comprehensive long-term follow-up with a multi-domain approach.[1 3 21-23]

The present study provides a deeper understanding of how the PSC structure could support patients in expressing their needs. Problems with communication and comprehension are common after stroke,[5] which influences decision-making during follow-up. Participation in decision-making requires health literacy, i.e., the ability to understand health information and a capacity to argue for one’s needs in relation to appropriate interventions.[7] In this study, despite perceptions that the PSC questions were easy to understand, dialogue was found to be crucial. Participants raised concerns about problem areas that could be missed depending on how the PSC was used. Results from using the PSC in the UK and Singapore[12] indicate that several problem areas could be indirectly identified. Awareness of the complexity of need identification underscores the role of the HP when using the PSC. Even if no unmet need is reported, a person can still identify as living with residual impairments and perceived problems in engaging in activities.[24] Participants expressed that identification of needs could be enhanced if more time were allowed for consideration of these needs and for additional questions; another help would be the opportunity to fill in the PSC beforehand. Current findings stress HP with stroke expertise and the need to make space for dialogue when administering the PSC to support needs identification and decision-making.

The results from the present study highlight the dialogue between the patient and HP, which is central in health care.[9 10 25] Experienced HPs argued that they could cover most topics using open-ended questions. In contrast, others emphasised the value of articulating the

For peer review only - http://bmjopen.bmj.com/site/about/guidelines.xhtml

PSC questions literally. To enable investigation of specific areas, closed questions can be important[26] and facilitate situations involving patients with communication difficulties. However, the PSC instructions do not hinder its use in a looser way as long as all areas are captured. The result shows that patients can benefit from a clear structure when the PSC is used. Participants in all focus groups agreed on the benefits of going through the areas in the PSC in a way that ensures identification of unmet needs. In addition, using the PSC in combination with dialogue supports the patients' capacity to communicate their needs. A narrative communication, along with signs of problems, gives the HP a foundation for planning care together with the patient and creates conditions for patients to make appropriate health decisions.[10]

The PSC can improve clinical pathways in health care by its structure and guidance for further referrals. Creating a plan to take care of identified needs and locally adapted pathways to support access to appropriate interventions, was noted in the focus group discussions as essential.

Strengths and limitations

A strength of this study is that patients were partners throughout the project, from the translation process to participation in the focus group discussions. In addition, the mix of methods made it possible to explore the feasibility of the PSC at different levels compared to previous research. Few persons in this population had lived with their stroke for a long time, and only one of them had a severe stroke, which might have affected which problems were identified (e.g. spasticity). This naturalistic design in an ordinary outpatient context, however, is representative of the Swedish population, where the majority have mild stroke.[27] The time (1-2 months) between the follow-up visit where the PSC was used and the focus group discussion could be a risk for recall bias. However, the focus group methodology enabled exploring a range of opinions of people across groups, and together, the participants contributed to rich discussions. Although the attempt was to obtain heterogeneity and homogeneity in the focus groups, the majority of HPs were women and the majority of the patients were male. Because the purposive sampling of HPs were made based on health care facilities already chosen and the defined time limit between the visit and the focus groups, the sex distribution were out of our influence. However, heterogeneity was achieved with respect to different outpatient settings, patients having a range of ages, stroke characteristics, and education levels, and HPs with a range of professional roles and experience in stroke. To strengthen the transferability of the findings, a comprehensive description of the study

For peer review only - http://bmjopen.bmj.com/site/about/guidelines.xhtml

context, participant characteristics, data collection, and analysis process are included in the methods section. Nevertheless, there are limitations regarding the transferability of the findings outside of the Swedish healthcare context. To ensure the feasibility of using the PSC in another context, a cross-cultural validation is needed. However, because the World Stroke Organization recommends using the PSC globally, these results contribute to a deeper understanding of its feasibility that can also be useful to other countries.

Conclusions

The PSC is a feasible and relevant tool to support egalitarian follow-up and identify patients who can benefit from targeted interventions after stroke. HPs' stroke expertise, room for dialogue, and caring for identified needs were raised as important issues to consider when using the PSC. Nutrition, sexuality, driving, work, vision, irritability and fatigue were areas mentioned that might need to be addressed within the discussions by HPs using the checklist. The PSC can facilitate patients in expressing their needs, enhancing their ability to participate in decision-making.

Acknowledgements

The authors thank the health professionals and patients, for sharing their experiences. The authors also thank Dr Kate Bramley-Moore translation help regarding quotations from the transcribed interviews.

Author contribution

EK, ÅL-N, and KSS contributed to the design of the study. EK conducted the interviews and analyzed the data together with GC, involving KSS and ÅL-N in the final stages of the analysis. EK wrote the first version of the manuscript, which was reviewed by GC, ÅL-N KSS. All four authors contributed to and approved the final manuscript.

Funding

The study was funded in part by the Swedish Stroke Association; the Local Research and Development Board for Gothenburg and Södra Bohuslän; the Swedish Heart and Lung Foundation; the Swedish Brain Foundation; an unconditional grant from Allergan; the Hjalmar Svenssons Foundation; Neurological Foundation; Greta and Einar Askers Foundation; Rune and Ulla Amlövs Foundation; Per-Olof Ahls Foundation; and John and Brit Wennerströms Foundation. The study was supported by grants from the Swedish state under

For peer review only - http://bmjopen.bmj.com/site/about/guidelines.xhtml

the agreement between the Swedish government and the county councils, the ALF agreement (ALFGBG-719 80).

Competing interests

None declared.

Data sharing

Due to ethical restrictions, data are available upon request. Researchers can submit requests for data to the authors (contact: ks.sunnerhagen@neuro.gu.se). Complete data from interviews cannot be made publicly available for ethical and legal reasons, according to the Swedish regulations http://www.epn.se/en/start/regulations/. Public availability would compromise participant privacy or confidentiality. Upon request a list of condensed meaning units or codes can be made available after removal of information that may risk the confidentiality of the participants. To access data please contact the first author: (emma.kjork@neuro.gu.se).

For peer review only

Figure 1. The steps included in the validation and cross-cultural adaptation of the Swedish version of the PSC including background work and current study.

Figure 2. Percentages of patients with identified problems in each Post-Stroke Checklist item (n=46).

Figure 3. Themes and subthemes derived from the focusgroup discussions with patients and health professionals regarding experiences of using the Post-Stroke Checklist.

For peer review only - http://bmjopen.bmj.com/site/about/guidelines.xhtml

REFERENCES

1. Crichton SL, Bray BD, McKevitt C, et al. Patient outcomes up to 15 years after stroke: survival, disability, quality of life, cognition and mental health. *J Neurol Neurosurg Psychiatry* 2016;87(10):1091-8. doi: 10.1136/jnnp-2016-313361

2. Ullberg T, Zia E, Petersson J, et al. Changes in functional outcome over the first year after stroke: an observational study from the Swedish stroke register. *Stroke* 2015;46(2):389-94. doi: 10.1161/STROKEAHA.114.006538

3. Ullberg T, Zia E, Petersson J, et al. Perceived Unmet Rehabilitation Needs 1 Year After Stroke: An Observational Study From the Swedish Stroke Register. *Stroke* 2016;47(2):539-41. doi: 10.1161/STROKEAHA.115.011670

4. Carlsson GE, Moller A, Blomstrand C. Consequences of mild stroke in persons <75 years -- a 1-year follow-up. *Cerebrovasc Dis* 2003;16(4):383-8. doi: 10.1159/000072561

5. Cumming TB, Marshall RS, Lazar RM. Stroke, cognitive deficits, and rehabilitation: still an incomplete picture. *Int J Stroke* 2013;8(1):38-45. doi: 10.1111/j.1747-4949.2012.00972.x

6. Levasseur M, Carrier A. Integrating health literacy into occupational therapy: Findings from a scoping review. *Scand J Occup Ther* 2012;19(4):305-14. doi: 10.3109/11038128.2011.588724

7. Mackey LM, Doody C, Werner EL, et al. Self-Management Skills in Chronic Disease Management: What Role Does Health Literacy Have? *Med Decis Making* 2016;36(6):741-59. doi: 10.1177/0272989X16638330 [published Online First: 2016/04/08]

8. Kirkevold M. The unfolding illness trajectory of stroke. *Disabil Rehabil* 2002;24(17):887-98. doi: 10.1080/09638280210142239

9. Tistad M, Ytterberg C, Tham K, et al. Poor concurrence between disabilities as described by patients and established assessment tools three months after stroke: a mixed methods approach. *J Neurol Sci* 2012;313(1-2):160-6. doi: 10.1016/j.jns.2011.08.038 [published Online First: 2011/09/23]

10. Ekman I, Swedberg K, Taft C, et al. Person-centered care—Ready for prime time. *Eur J Cardiovasc Nurs* 2011;10(4):248-51.

11. Philp I, Brainin M, Walker MF, et al. Development of a poststroke checklist to standardize follow-up care for stroke survivors. *J Stroke Cerebrovasc Dis* 2013;22(7):e173-80. doi: 10.1016/j.jstrokecerebrovasdis.2012.10.016

For peer review only - http://bmjopen.bmj.com/site/about/guidelines.xhtml

12. Ward AB, Chen C, Norrving B, et al. Evaluation of the Post Stroke Checklist: a pilot study in the United Kingdom and Singapore. *Int J Stroke* 2014;9 Suppl A100(A100):76-84. doi: 10.1111/ijs.12291
13. Iosa M, Lupo A, Morone G, et al. Post Soft Care: Italian implementation of a post-stroke checklist software for primary care and identification of unmet needs in community-dwelling patients. *Neurol Sci* 2017;39(1):135-39. doi: 10.1007/s10072-017-3140-1
14. Hotter B, Padberg I, Liebenau A, et al. Identifying unmet needs in long-term stroke care using in-depth assessment and the Post-Stroke Checklist – The Managing Aftercare for Stroke (MAS-I) study. *European Stroke Journal* 2018;3(3):237-45. doi: 10.1177/2396987318771174
15. Krueger RA. Focus groups: A practical guide for applied research. 5th ed. London: Sage publications 2014.
16. Ivanoff SD, Hultberg J. Understanding the multiple realities of everyday life: basic assumptions in focus-group methodology. *Scand J Occup Ther* 2006;13(2):125-32.
17. Craig P, Dieppe P, Macintyre S, et al. Developing and evaluating complex interventions: the new Medical Research Council guidance. *BMJ* 2008;337:a1655. doi: 10.1136/bmj.a1655
18. Beaton DE, Bombardier C, Guillemin F, et al. Guidelines for the process of cross-cultural adaptation of self-report measures. *Spine (Phila Pa 1976)* 2000;25(24):3186-91. [published Online First: 2000/12/22]
19. Tong A, Sainsbury P, Craig J. Consolidated criteria for reporting qualitative research (COREQ): a 32-item checklist for interviews and focus groups. *Int J Qual Health Care* 2007;19(6)
20. Malterud K, Siersma VD, Guassora AD. Sample Size in Qualitative Interview Studies: Guided by Information Power. *Qual Health Res* 2015 doi: 10.1177/1049732315617444 [published Online First: 2015/11/29]
21. Paul SL, Srikanth VK, Thrift AG. The large and growing burden of stroke. *Curr Drug Targets* 2007;8(7):786-93. [published Online First: 2007/07/17]
22. Stevens E, Emmett E, Wang DY, et al. The Burden Of Stroke In Europe Report London: SAFE Stroke Alliance For Europe 2017.
23. Norrving B, Barrick J, Davalos A, et al. Action Plan for Stroke in Europe 2018–2030. *European Stroke Journal*;0(0):2396987318808719. doi: 10.1177/2396987318808719

For peer review only - http://bmjopen.bmj.com/site/about/guidelines.xhtml

24. Shannon RL, Forster A, Hawkins RJ. A qualitative exploration of self-reported unmet need one year after stroke. *Disabil Rehabil* 2016;38(20):2000-7. doi: 10.3109/09638288.2015.1107784

25. Williams MV, Davis T, Parker RM, et al. The role of health literacy in patient-physician communication. *Fam Med* 2002;34(5):383-9.

26. Silverman J, Kurtz S, Draper J. Skills for communicating with patients. 3rd ed. London: CRC Press 2016.

27. Reinholdsson M, Palstam A, Sunnerhagen KS. Prestroke physical activity could influence acute stroke severity (part of PAPSIGOT). *Neurology* 2018;91(16):e1461-e67. doi: 10.1212/WNL.0000000000006354 [published Online First: 2018/09/21]

For peer review only

For peer review only - http://bmjopen.bmj.com/site/about/guidelines.xhtml

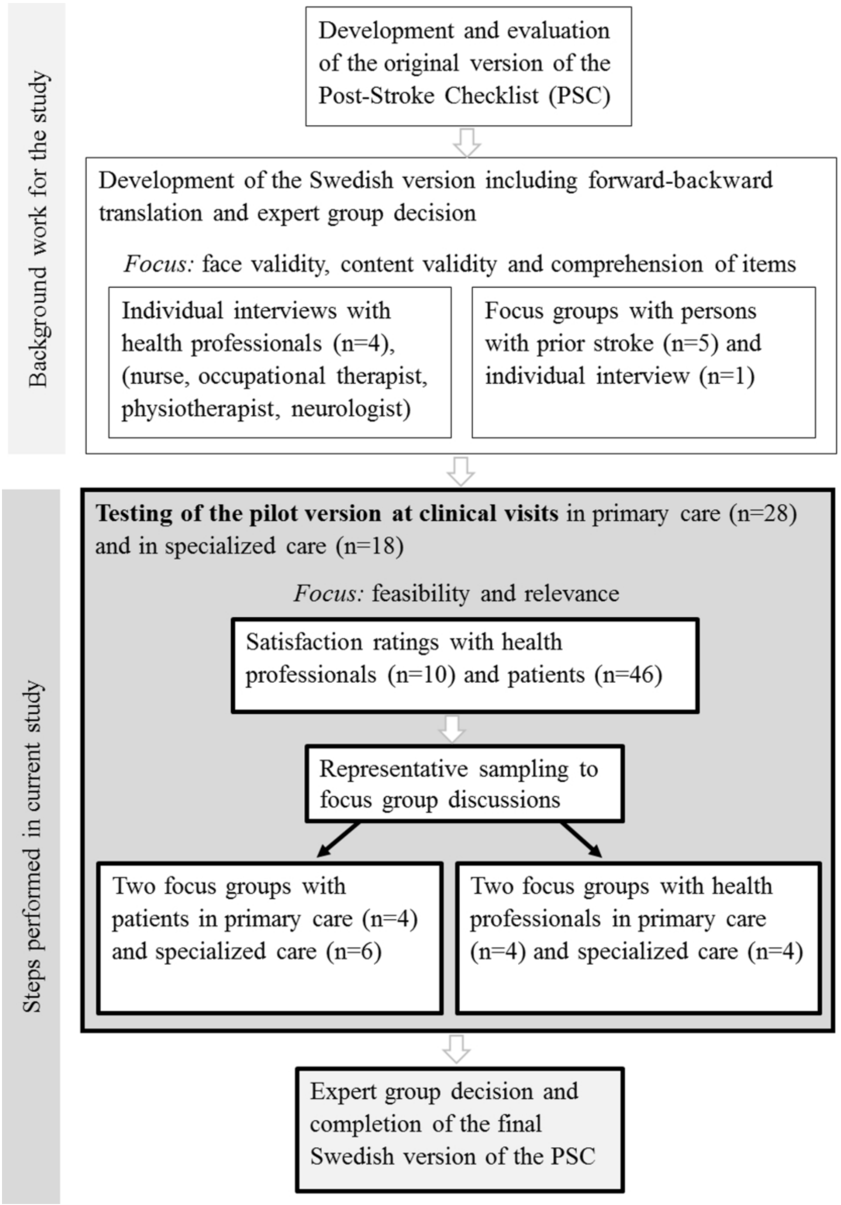

Figure 1. The steps included in the validation and cross-cultural adaptation of the Swedish version of the PSC including background work and current study.

199x267mm (300 x 300 DPI)

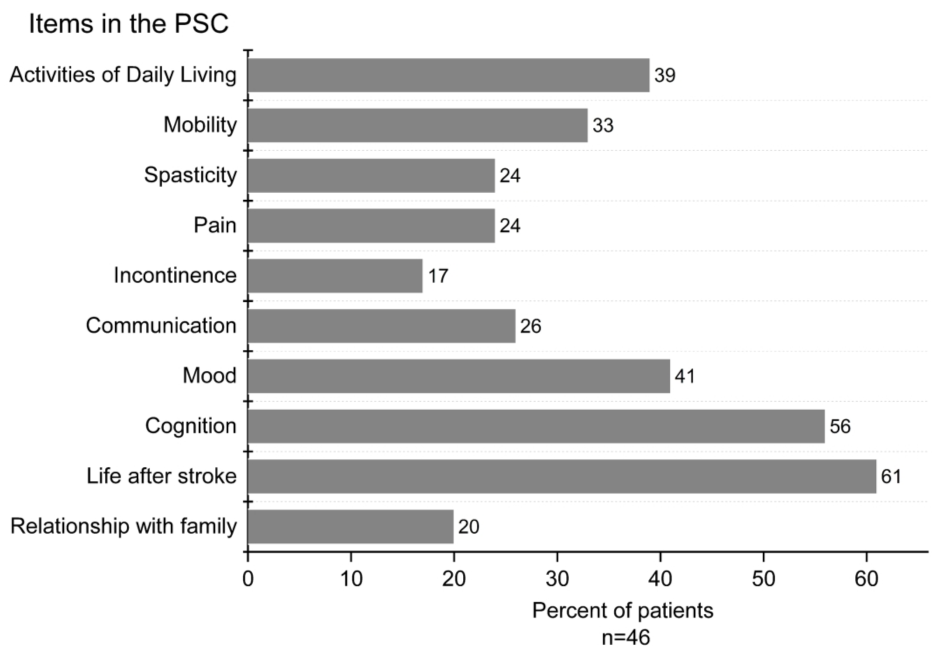

Figure 2. Percentages of patients with identified problems in each Post-Stroke Checklist item (n=46).

229x160mm (300 x 300 DPI)

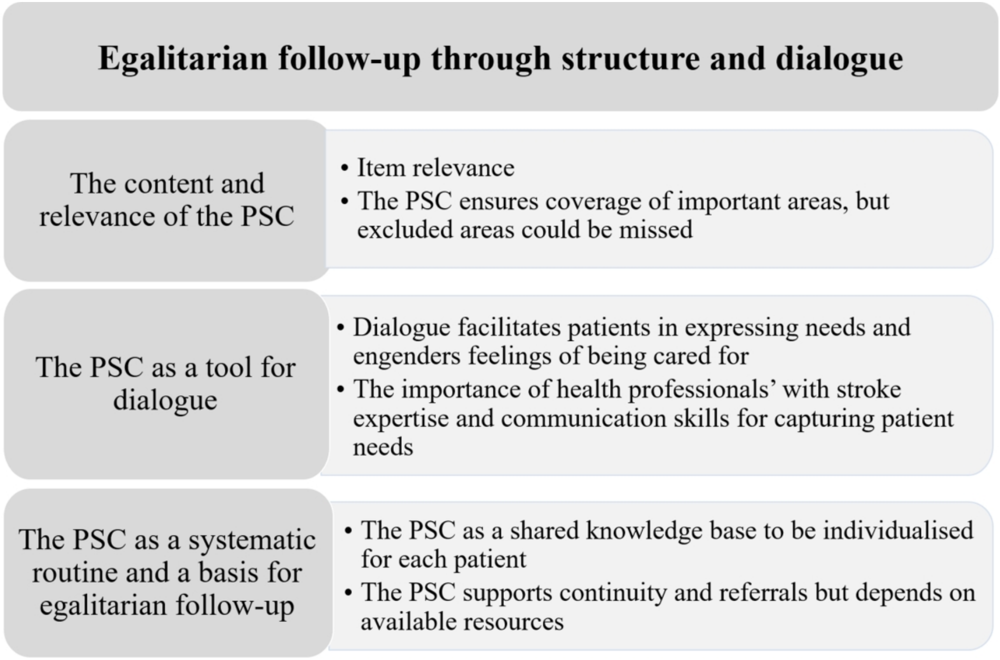

Figure 3. Themes and subthemes derived from the focusgroup discussions with patients and health professionals regarding experiences of using the Post-Stroke Checklist.

169x111mm (300 x 300 DPI)

COREQ 32-item checklist

**No. Item**	**Guide questions/description**	**Comments**
**Domain 1: Research team and reflexivity**		
Personal characteristics		
1. Interviewer/facilitator	Which author/s conducted the interview?	EK (p.6)
2. Credentials	What were the researcher's credentials? *e.g., PhD, MD*	3 Occupational Therapists (1 associate professor, 1 PhD, and 1 MS, PhD student)<br>1 Medical doctor, (Professor)
3. Occupation	What was their occupation at the time of the study?	Researchers
4. Gender	Was the researcher male or female?	4 female (p. 6-7)
5. Experience and training	What experience or training did the researchers have?	> 20 years in stroke rehab, Qualitative research (p. 7)
Relationship with participants		

For peer review only

6. Relationship established	Was a relationship established prior to study commencement?	The moderator had met some of the health professionals in work related situations.
7. Participant knowledge of the interviewer	What did the participants know about the researcher? *e.g., personal goals, reasons for doing the research*	Broad outlines given
8. Interviewer characteristics	What characteristics were reported about the interviewer/facilitator? *E.g., bias, assumptions, reasons and interests in the research topic*	(p. 5)
**Domain 2: study design**		
Theoretical framework		
9. Methodological orientation and Theory	What methodological orientation was stated to underpin the study? *e.g. grounded theory, discourse analysis, ethnography, phenomenology, content analysis*	Focus group according to Kreuger, underpinned by a methodology based on Social constructivism (p. 4)
Participant selection		

For peer review only

10. Sampling	How were participants selected? *e.g. purposive, convenience, consecutive, snowball*	Purposive (p. 5)
11. Method of approach	How were participants approached? e.g. *face-to-face, telephone, mail, email*	Face to face and by telephone (p. 5)
12. Sample size	How many participants were in the study?	In total, 18 (patients/HPs) participated in the focus groups (see table 2, p 7)
13. Non-participation	How many people refused to participate or dropped out? Reasons?	One dropped out because of a scheduled medical examination (p. 9)
Setting		
14. Setting of data collection	Where was the data collected? *e.g. home, clinic, workplace*	Clinical setting (p. 5)
15. Presence of non-participants	Was anyone else present besides the participants and researchers?	No (p. 6)
16. Description of sample	What are the important characteristics of the sample? *e.g. demographic data, date*	People with stroke in a late phase of stroke recovery (p. 7, table 2)
Data collection		

For peer review only

17. Interview guide	Were questions, prompts, guides provided by the authors? Was it pilot tested?	A interview guide was provided (p.6). A pilot testing was conducted (p. 5).
18. Repeat interviews	Were repeat interviews carried out?	No, each group met once (p. 6)
19. Audio/visual recording	Did the researchers use audio or visual recording to collect the data?	Audio (p. 6)
20. Field notes	Were field notes made during and/or after the interview or focus group?	Yes
21. Duration	What was the duration of the interviews or focus group?	Approximately 1.5 hours (p. 6)
22 Data saturation	Was data saturation discussed?	Yes (p. 6)
23. Transcripts returned	Were transcripts returned to participants for comment and/or correction?	No
**Domain 3: analysis and findings**		
Data analysis		
24. Number of data coders	How many data coders coded the data?	Two (EK and GC) (p. 6)
25. Description of the coding tree	Did authors provide a description of the coding tree?	Yes.
26. Derivation of themes	Were themes identified in advance or derived from the data?	Derived from the data (p. 6)

For peer review only

27. Software	What software, if applicable, was used to manage the data?	Nvivo (p. 6)
28. Participant checking	Did participants provide feedback on the findings?	No, not specifically, but a general presentation (p. 5)
Reporting		
29. Quotations presented	Were participant quotations presented to illustrate the themes/findings? Was each quotation identified? *e.g. participant number*	Yes (Results)
30. Data and findings consistent	Was there consistency between the data presented and the findings?	Yes (Results and Discussion)
31. Clarity of major themes	Were major themes clearly presented in the findings?	Yes (Figure 3 and results)
32. Clarity of minor themes	Is there a description of diverse cases or discussion of minor themes?	Yes (Results and Discussion)

For peer review only
